# Genome analysis of deep sea piezotolerant *Nesiotobacter exalbescens* COD22 and toluene degradation studies under high pressure condition

**DOI:** 10.1038/s41598-019-55115-9

**Published:** 2019-12-10

**Authors:** Ganesh Kumar A., Noelin Chinnu Mathew, Sujitha K., Kirubagaran R., Dharani G.

**Affiliations:** Marine Biotechnology Division, Earth System Science Organization - National Institute of Ocean Technology (ESSO – NIOT), Ministry of Earth Sciences (MoES), Government of India, Pallikaranai, Chennai, 600100 India

**Keywords:** Water microbiology, Pollution remediation

## Abstract

A marine isolate, *Nesiotobacter exalbescens* COD22, isolated from deep sea sediment (2100 m depth) was capable of degrading aromatic hydrocarbons. The *Nesiotobacter* sp. grew well in the presence of toluene at 0.1 MPa and 10 MPa at a rate of 0.24 h^−1^ and 0.12 h^−1^, respectively, in custom designed high pressure reactors. Percentage of hydrocarbon degradation was found to be 87.5% at ambient pressure and it reached 92% under high pressure condition within a short retention period of 72 h. The biodegradation of hydrocarbon was confirmed by the accumulation of dicarboxylic acid, benzoic acid, benzyl alcohol and benzaldehyde which are key intermediates in toluene catabolism. The complete genome sequence consists of 4,285,402 bp with 53% GC content and contained 3969 total coding genes. The complete genome analysis revealed unique adaptation and degradation capabilities for complex aromatic compounds, biosurfactant synthesis to facilitate hydrocarbon emulsification, advanced mechanisms for chemotaxis and presence of well developed flagellar assembly. The genomic data corroborated with the results of hydrocarbon biodegradation at high pressure growth conditions and confirmed the biotechnological potential of *Nesiotobacter* sp. towards bioremediation of hydrocarbon polluted deep sea environments.

## Introduction

Oil spill accidents and pollutant accumulation in coastal and deep sea environment have been of great concern. In United States, Refugio oil spill discharged more than 100,000 gallons of crude oil into the marine environment in the year 2015 (https://calspillwatch.dfg.ca.gov). In the ocean, oil spills containing hydrocarbon mixtures released on the surface water get widely dispersed and trickle into different depths and end up in sediment layers based on water currents. Mixture of oil and particulate aggregates constitutes for the distribution as well as accumulation of the soluble and insoluble hydrocarbons in marine sediments^1^. The persistence nature and refractory property restrict the rate of degradation process^2^. Toluene, a monoaromatic hydrocarbon, produced by petrochemical plants/petroleum refineries has a large number of commercial applications. In industrial sector, toluene is utilized as a raw material in chemical synthesis, manufacture of polymers, dyes, paints and adhesives. The toxic nature of BTEX (benzene, toluene, ethylbenzene and xylene) and polycyclic aromatic hydrocarbons (PAHs) causes serious threat to ecosystem. In marine and groundwater environment, BTEX are the major compounds causing contamination. These hydrocarbons generate serious health effects on living organisms^3^. It enters the marine environment during transportation and accidental spillage. Although bioaccumulation in marine organisms is reported to be low, bioaccumulation of 2, 4, 6-trinitrotoluene (TNT), a derivative of toluene, have been observed in marine fishes^4^. Based on toxic potential and environmental contamination of toluene derivatives, improved bioremediation technologies are required imminently. Bioremediation application proves to be a potent eco-friendly option to treat highly polluted marine environment^5^. The piezotolerant marine strains have the ability to function at both the low and high pressure conditions; bioremediation of toluene can be carried out using hydrocarbonoclastic microorganisms having the ability to degrade hydrocarbons. Information available about such microorganisms is limited or perhaps nil to our knowledge. Previously, we reported the potentialities of piezotolerant bacteria in breaking down complex hydrocarbons^6^. Similarly, *Rhodococcus qingshengii* TUHH-12 was reported to be capable of degrading *n*-hexadecane at 15 MPa in high-pressure reactors^7^. A report by Cui *et al*., revealed that *Cycloclasticus* isolated from Atlantic sediment at a depth of 3542 km was vital in degrading PAHs^8^. A PAHs utilizing bacterium *Celeribacter indicus* sp. nov., was isolated from deep sea sediment of the Indian Ocean^9^. These significant findings show that the pattern and type of degradation depends much on the nature of microorganisms and its ecosystem. Unlike terrestrial, not many marine strains have been studied for its ability to degrade toluene and interestingly no report is available on growth and hydrocarbon degradation pattern under ambient and elevated pressure conditions. Studies on hydrocarbonoclastic marine bacteria capable of degrading hydrocarbons at high pressure conditions are very limited.

Several studies have reported the complete genome sequence of hydrocarbon degrading bacteria which contribute to understanding of multiple networks established during hydrocarbon degradation^10–12^. The complete genome analysis further specify systems for genetic adaptations of the strains, uptake mechanisms of nutrients, production of bio-surfactants and response to stressed environmental conditions. Reports exist on complete genome of marine oil degrading bacteria, however, research on the metabolic pathway and role of genetic mechanism during hydrocarbon degradation processes in deep sea bacteria is scanty. Only few studies are available on the ability of deep sea bacteria to degrade hydrocarbons in high pressure environment. No information is available on *Nesiotobacter* sp. from deep sea environment till date. This work emphasizes the role of a hydrocarbonoclastic deep sea bacterial strain in the degradation processes of toluene at *in situ* deep sea conditions. Additionally, we report here the whole genome sequence of the deep sea, piezotolerant, hydrocarbonoclastic bacterium *Nesiotobacter exalbescens*. The genetic study on the deep sea bacterium was carried out because of its ability to degrade PAHs and tolerance to elevated pressure conditions. A genetic study was essential in this species in order to identify its functional competence for (i) aromatic hydrocarbon degradation and metabolism (ii) biosurfactant production and (iii) chemotaxis. Our study assumes great importance by the way of exploring its significant growth at elevated pressure, a distinct pathway during biodegradation process and involvement of novel genes in biotransformation of hydrocarbons.

## Results

### Isolation and identification of *Nesiotobacter* sp

**Figure 1:**
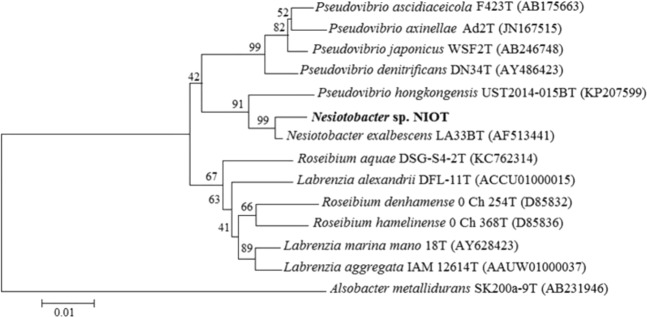
Neighbour-Joining phylogenetic tree based on the 16S rDNA sequence of *Nesiotobacter* sp. *Alsobacter metallidurans* SK200a-9^T^ was used as out-group. Bootstrap (%) based on the NJ analysis of 1000 resampled datasets were indicated as numbers at nodes. Score bar represents 0.01 substitutions per site.

A strain NIOT COD22 isolated from 2100 m deep sea sediment sourced from the Bay of Bengal was capable of degrading toluene. The cell size ranged from 2–2.5 µm, gram negative short rods, motile, oxidase positive, catalase negative and nitrate positive. Analysis of carbohydrates, amino acids and organic acids utilization revealed that strain was able to utilize the following wide range of substrates: maltose, fructose, mannose, sorbose, ornithine, phenyl alanine and lysine. Several other sources were negative, namely, adonitol, α-methyl D-mannoside, α-methyl D-glucoside, cellobiose, dextrose, dulcitol, D-arabinose, erythritol, esculin, galactose, glycerol, inulin, inositol, lactose, L-arabinose, melibiose, mannitol, malonate, melezitose, rhammnose, raffinose, sucrose, sodium gluconate, salicin, sorbitol, trehalose, xylose and xylitol. The biochemical tests were negative for H_2_S production, citrate, ortho-nitrophenyl-β-galactoside (ONPG) and urease. The 16S rRNA sequencing for this isolate is around 1200 nucleotides which exhibited 83% similarity. Neighbour-joining analysis from MEGA6 was employed for picturing the phylogenetic tree pattern, henceforth, depicted the relationship of this marine isolate to its closely related taxa. Phylogenetic tree depicted the close relatedness of NIOT COD22 strain to *Nesiotobacter exalbescens* (Fig. 1). The sequence of the 16S rRNA gene obtained was deposited in the EMBL under accession number LK054394. The genomic sequence has an average G + C content of 53 mol%.

### Bacterial growth physiology

The growth of *N*. *exalbescens* COD22 at different pH, salt concentration and temperature variations was studied for possible bioremediation applications in challenging marine environment. The isolate grew at 10–40 °C with maximum growth at 28 °C.

In terms of salinity, growth was observed at various concentrations of NaCl ranging from 1–8% (w/v) with an optimum at 4% (w/v). This species was found to grow within a pH range of 6.0–10.0 with an optimum growth at pH 7.0.

### Substrate specificity of isolate

Different functional characteristics of deep sea isolate were examined for multiple substrate utilization. The substrate specificity test (Table 1) revealed that the isolate grew profusely on the tested substrates such as brij-35, cedar wood oil, crude oil, diesel, hexadecane, kerosene, petrol, phenol, sodium dodecyl sulphate, spent engine oil, toluene, triton X-100, tween 80 and xylene. However, growth was slow in silicone oil, while no growth was observed in presence of clove oil.

**Table 1:** Substrates specificity of *Nesiotobacter* sp.

Substrate	Growth
Brij-35	+++
Cedar wood oil	++
Clove oil	−
Crude oil	+++
Diesel	+++
n-Hexadecane	+++
Kerosene	+++
Petrol	+++
Phenol	+
Silicone oil	+
Sodium dodecyl sulphate	++
Spent Engine oil	++
Toluene	+++
Triton X-100	+++
Tween 80	+++
Xylene	+++

(−) indicates isolate could not use substrate as carbon source, (+) indicates poor growth on substrate, (++) indicates good growth on substrate, (+++) indicates luxuriant growth and isolate could use substrate effectively as carbon source.

### Degradation of toluene by *N*. *exalbescens* COD22 at ambient and high pressure

**Figure 2:**
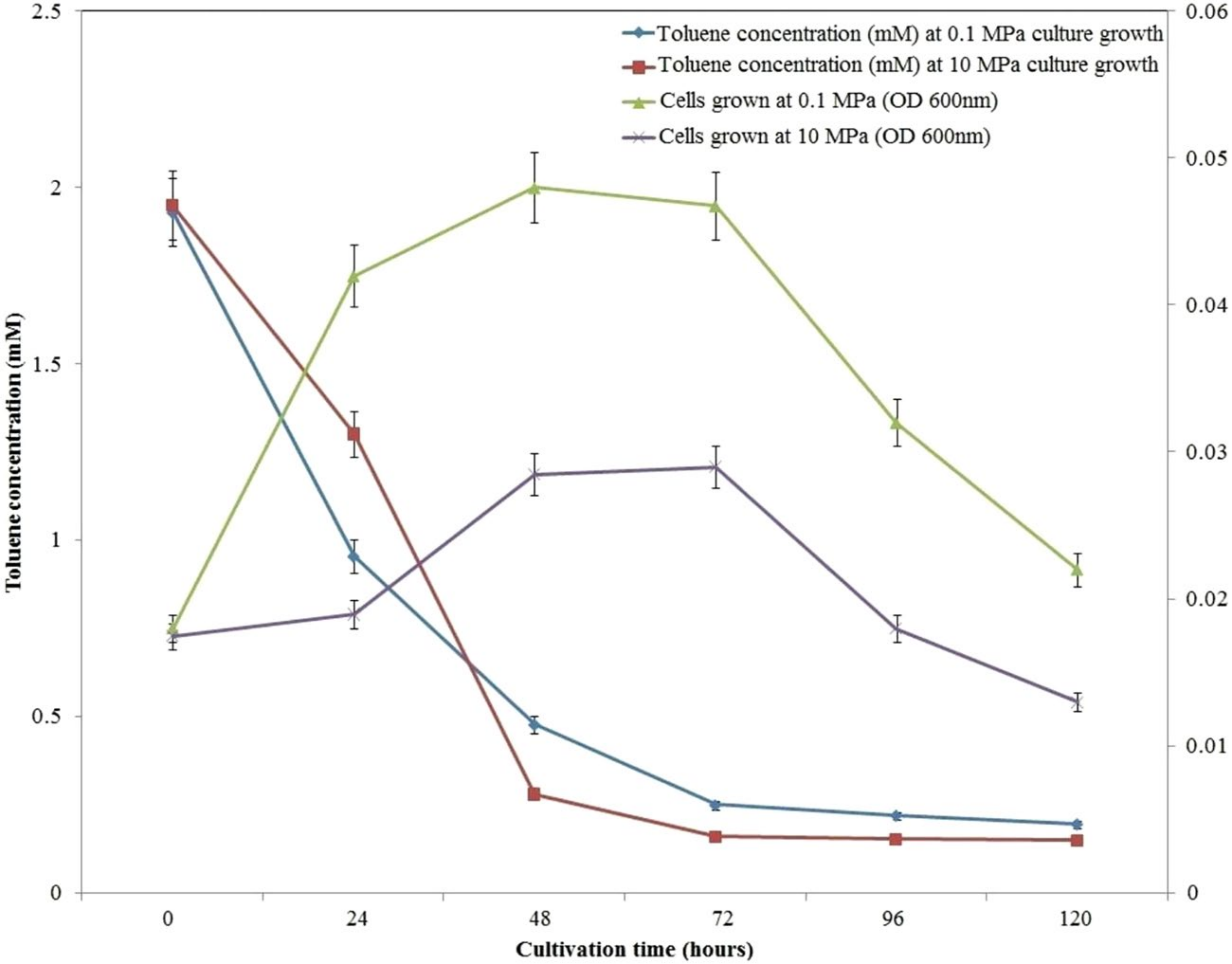
Degradation of toluene and growth of *N*. *exalbescens* COD22 at different cultivation period under 0.1 MPa and 10 MPa pressure conditions.

The suspended bacterial cells were grown on toluene as sole carbon source at 0.1 MPa and 10 MPa pressure conditions. In order to evaluate the effect of high pressure on the isolate and to simulate the physical conditions encountered at 1000 m depth below ocean surface; the minimal salt medium was amended with toluene and incubated for different time intervals at 10 MPa pressure and 20 °C temperature in the high pressure reactor. The cells grew well when glucose was given as sole carbon source at 0.1 MPa and 10 MPa pressure conditions. In 10 MPa pressure condition, an increase in cell count was observed between 24 h and 48 h, and the total cells increased from 5 × 10^3^ to 1.85 × 10^5^ cells/mL. This result confirmed that the growth of this species was independent of pressure. Biodegradation of toluene by *N*. *exalbescens* COD22 is shown in Fig. 2. The isolate grew well and degraded the aromatic hydrocarbon at both pressure conditions (0.1 MPa and 10 MPa) tested.

### SEM analysis of piezotolerance in *N*. *exalbescens* COD22

**Figure 3:**
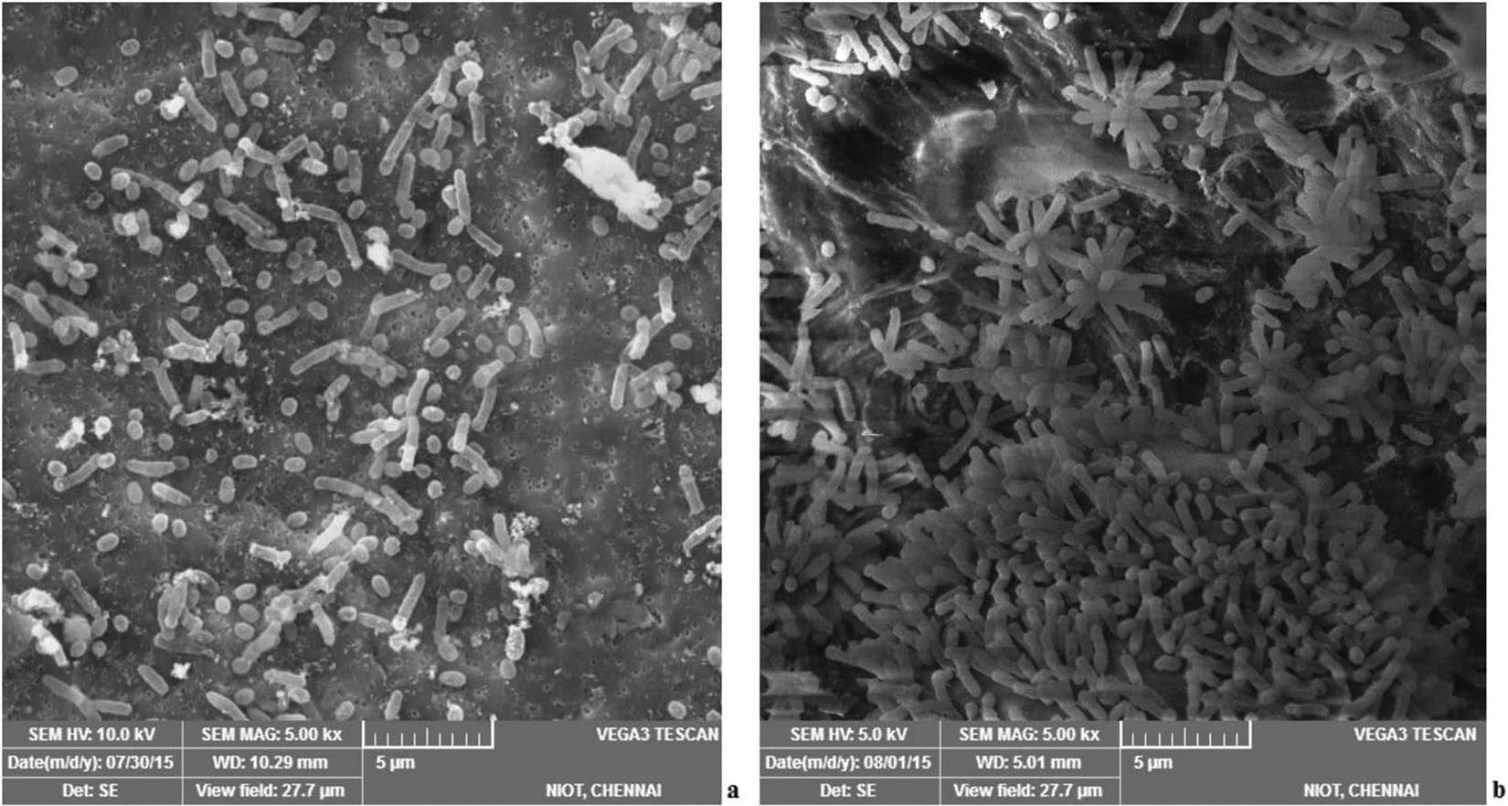
*N*. *exalbescens* COD22 observed during degradation of toluene by SEM (500×). (**a**) free cells at 0.1 MPa, (**b**) cell clusters at 10 MPa.

The cells were collected before and after exposure to high pressure treatments. The bacterial cells were rod shaped, 0.3–0.6 mm wide by 1.0–3.5 mm long (Fig. 3a). Interestingly, all the cells grown at 10 MPa pressure appeared to aggregate together in the growth phase (Fig. 3b).

### FTIR characterization of toluene degradation

**Figure 4:**
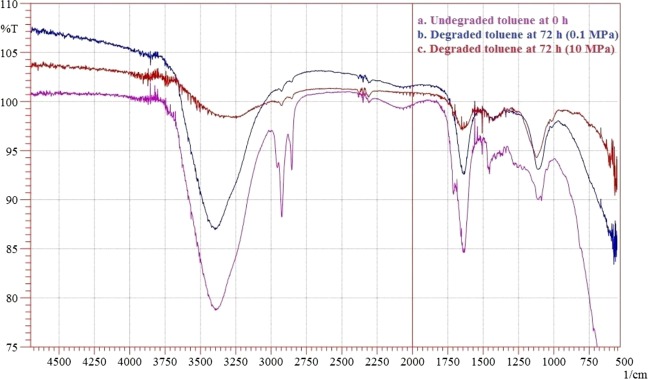
FTIR spectra analysis (**a**) toluene before degradation, (**b**) toluene degradation at 0.1 MPa and (**c**) toluene degradation at 10 MPa pressure conditions.

The degradation of toluene by *N*. *exalbescens* COD22 in 0.1 and 10 MPa growth conditions at optimized period of 72 h was confirmed by analyzing the breakdown and converted metabolites using FTIR spectroscopy. The FTIR spectrum obtained on 0^th^ day before the onset of degradation showed peaks at 3384, 3392, 3420 and 3445 cm^−1^ which corresponds to the NH and OH stretch of primary amines and alcohols in the toluene. The distinct peak appearing at 2925 cm^−1^ was attributed to the –C–H stretching of the alkyl methyl groups. Peak at 1640 cm^−1^ corresponds to the C=C stretch of alkenes. Many peaks between 1400–1500 cm^−1^ and 1575–1670 cm^−1^ were due to C–C stretches in the aromatic ring. The C–H in-plane deformation was obtained as weak band at 1250 cm^−1^ (Fig. 4a). In the toluene degradation at ambient pressure conditions, the spectrum is mainly characterized by broad band peak at 1100 cm^−1^ and this corresponds to the C–O stretching vibrations of primary alcohol, carboxylic and ester groups (Fig. 4b). In 10 MPa culture conditions, few bands showed less intensity and some bands became strong due to by-product formations and degradation of compounds (Fig. 4c).

### GC-MS analysis of toluene biodegradation

The conversion of toluene into its breakdown metabolites at 0.1 MPa and 10 MPa were analyzed using GC-MS at different retention time (Table 2). The peak at *Rt* 39.43 min corresponds to undegraded toluene samples. The *N*. *exalbescens* COD22 was able to completely degrade toluene within 72 h at 0.1 MPa pressure. Following treatment with our isolate, the metabolites eluted at retention time (*Rt*) of 39.4 and 40.3 min corresponds to the breakdown products, such as phenol, 2, 4,6-tris (1-methyle) and benzoic acid, 4-ethoxy-,eth, respectively. Cleavage of toluene can take place symmetrically or asymmetrically. Metabolization by meta- and ortho-pathways consecutively led to the accumulation of ring-cleavage metabolites in the medium during growth^13^. The presence of 1, 4-benzenediamine at *Rt* (60.452) in 96 h may be the outcome of hydroxylation and ring cleavage. Interestingly, the aromatic benzene compounds and its derivatives were effectively degraded in 120 h and elucidated by the accumulation of benzoic acid, 4-ethoxy at *Rt* (40.324) and 1, 2-benzenedicarboxylic acid at *Rt* (55.622), respectively. The accumulation of carboxylic acids corroborated with the research findings of Sharma and Pant (2000)^14^. At 10 MPa culture conditions, the major end product benzyl alcohol (*Rt* 17.902) was observed within 72 h of incubation (Table 2). The metabolites for both aerobic and anaerobic catabolism have been found in the samples tested.

**Table 2:** Compounds identified by GC-MS in toluene degradation at different pressure conditions.

Free cells	Products	RT	Time period
0.1 MPa	Butylated hydroxytoluene	39.43	24 h
	Butylated hydroxytoluene	39.43	72 h
	Phenol, 2, 4,6-tris (1-methyle)	39.435	72 h
	Benzoic acid, 4-ethoxy-,eth	40.328	72 h
	Phenol, 3,4,5-trimethoxy-	43.585	96 h
	Benzaldehyde, 4-hydroxy-3,5-	45.382	96 h
	1,4-Benzenediamine, N-(1-met	60.452	96 h
	Benzoic acid, 4-ethoxy-,eth	40.324	120 h
	1,2-benzenedicarboxylic acid	55.622	120 h
10 MPa	Butylated hydroxytoluene	39.43	24 h
	3-Ethoxy-4-methoxyphenol	47.284	24 h
	1,2-benzenedicarboxylic acid	55.62	24 h
	1, 3-Benzenediol, 5–pentadecy	84.035	24 h
	3-Ethoxy-4-methoxyphenol	47.320	48 h
	Benzyl alcohol	17.902	72 h
	Benzenepropanoic acid	32.503	72 h
	Benzoic acid, 4-ethoxy-,eth	40.328	72 h

### Whole genome sequencing and bioinformatics analysis

**Figure 5:**
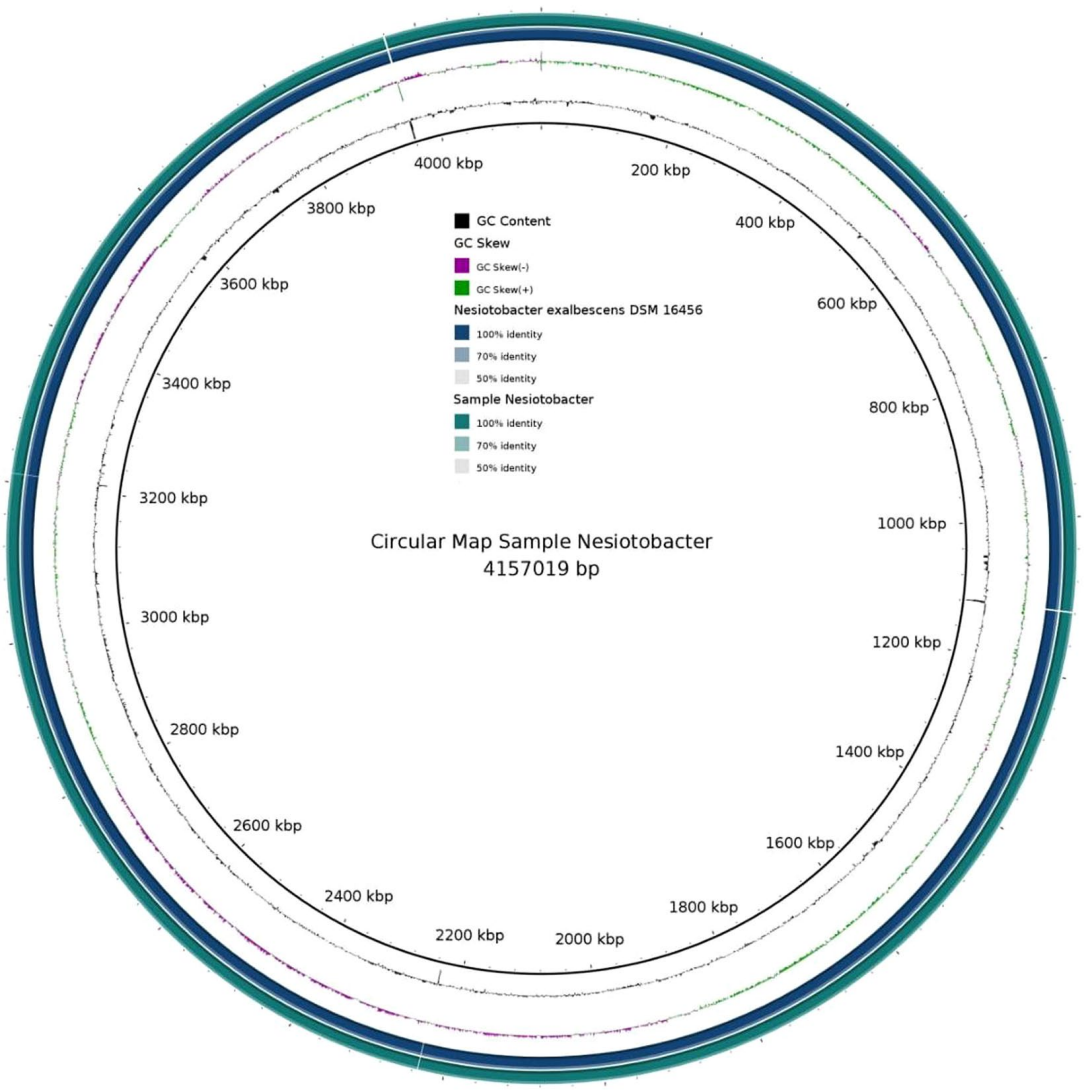
Circular representation of the single circular chromosome of *N*. *exalbescens* COD22 with homologs in *Nesiotobacter exalbescens* DSM 16456 (dark blue).

**Figure 6:**
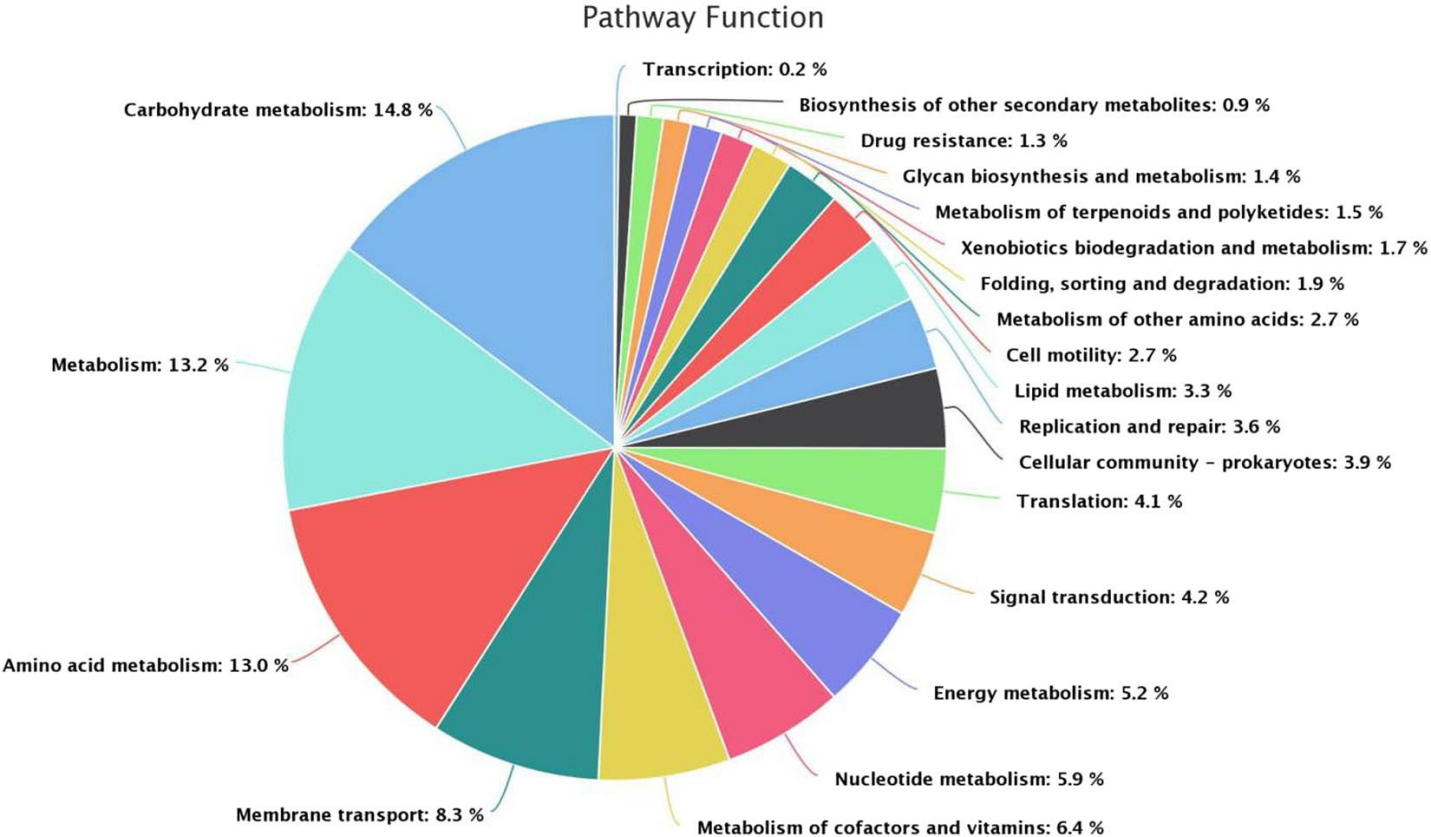
Clusters of orthologous groups (COG) of *N*. *exalbescens* COD22 confirming higher gene abundance for biodegradation of xenobiotics and metabolism, cell motility, lipid metabolism, metabolism of terpenoids and biosynthesis of other secondary metabolites.

The validation of the whole genome sequence was confirmed by a complete bacterial chromosome map (BAC) as given in Fig. 5. The image was generated using BRIG-v0.95 tool and homology was performed using the back end tool in NCBI-BLAST −2.2.29. The genome of *N*. *exalbescens* COD22 is comprised of a single circular chromosome of 4,285,402 base pairs (bp) that were assembled in multiple scaffolds (92 contigs) with an average G + C content of 53%. A total of 3969 coding genes were indentified of which, functions were assigned to 3553 genes and 416 genes were of unknown function. The general features of the genome are given in Table 3. The whole genome shot gun project has been deposited at Gen Bank under the accession number NKQT00000000. The detailed profiling of clusters of orthologous groups (COG) analysis provides in-depth details into the genomic capability of this isolate for degradation and survival in PAHs contaminated complex habitat (Fig. 6.) Metabolism forms the major function viz. carbohydrate, amino acid, cofactors and vitamins, nucleotide and energy with 14.8%, 13%, 6.4%, 5.9% and 5.2% respectively (Fig. 6). However, other functions such as xenobiotics and biodegradation (1.7%) and metabolism of terpenoids and polyketides (1.5%) are of particular interest from the point of view of this study.

**Table 3:** General features of the *N*. *exalbescens* COD22 genome.

Features	Description
Size (bp)	4,285,402
G + C% content	53%
Total coding genes	3969
Function assigned	2971
Uncharacterized protein	582
Putative uncharacterized protein	79
Function unknown	416
N50 value	1,043,397
Mean sequence length, bp	46583.29 ± 181634.65
Minimal length, bp	128
Maximal length, bp	11471860
Minimum GC content	27%
GC content range	74%
Maximum percentage of Ns per sequence	5%
rRNA	6
tRNA	63

### Degradation of crude oil components

**Figure 7:**
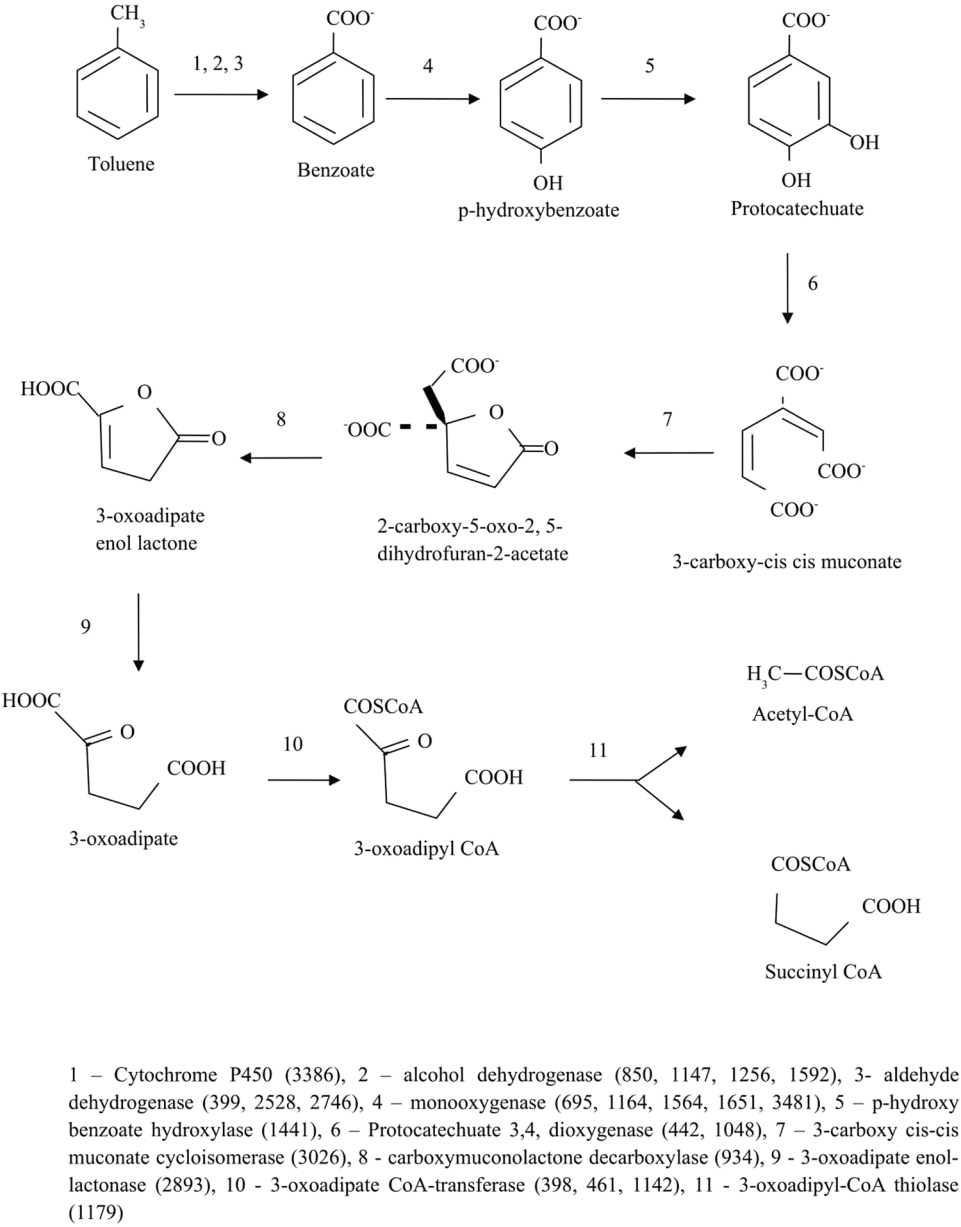
Proposed aerobic pathway during toluene degradation in *N*. *exalbescens* COD22.

**Figure 8:**
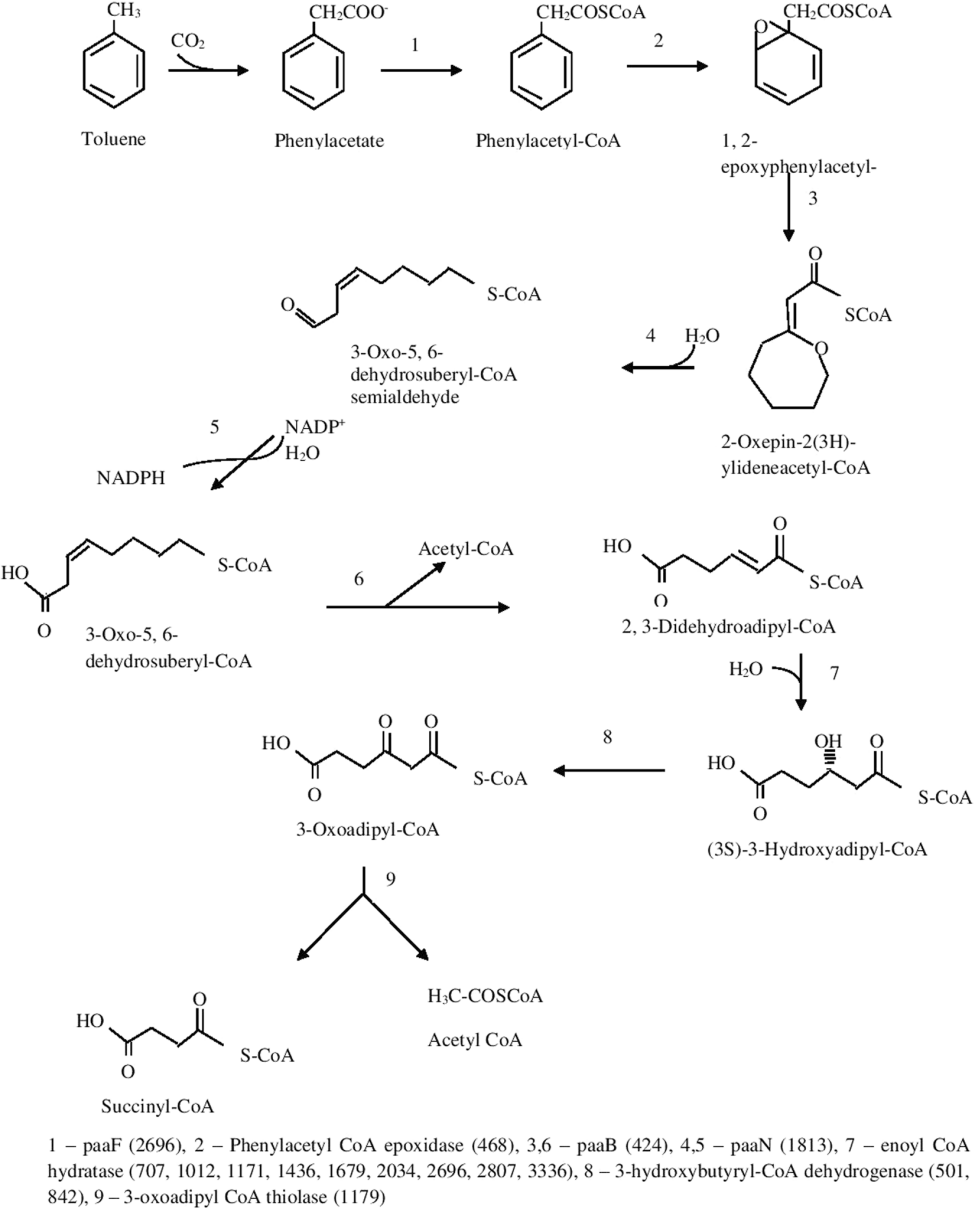
Proposed anaerobic pathway during toluene degradation in *N*. *exalbescens* COD22.

Genes responsible for degradation of oil components, including PAHs, biphenol, naphthalene, toluene, benzene and styrene were identified in the genome of *N*. *exalbescens* COD22: NKQT00000000 (Supplementary File). It is interesting to note that this strain is facultative anaerobe, which was identified by the whole genome analysis which revealed the presence of genes responsible for aerobic and anaerobic toluene degradation. The aerobic degradation pathway of toluene and enzymes involved in biotransformation are shown in Fig. 7. In aerobic pathway the initial oxidation of the methyl-group of toluene was catalyzed by a membrane-bound cytochrome P450 which was found to be down regulated during toluene degradation. Followed by the action of dehydrogenases, oxidized toluene was converted to benzoate. It was observed that the two putative enzymes involved in degradation were located concurrently in genomic assembly and hence it can be either up regulated or down regulated. The conversion of benzoate to *p-*hydroxybenzoate by monooxygenase was upregulated in the toluene experiment. *p-*Hydroxybenzoate was oxidized to protocatechuate by *p-*hydroxybenzoate hydroxylase (PHBH). In protocatechuate, the aromatic ring was opened by the dioxygenase (P340) to form 3-carboxy cis cis muconate and was further processed by 3-carboxy-cis, cis-muconate cycloisomerase to 2-carboxy-5-oxo-2,5-dihydrofuran-2-acetate. Meanwhile, 2-carboxy-5-oxo-2,5-dihydrofuran-2-acetate was processed simultaneously by carboxymuconolactone decarboxylase to 3-oxoadipate enol-lactone. Following this, 3-oxoadipate enol-lactonase further converts 3-oxoadipate enol-lactone to 3-oxoadipate. 3-Oxoadipate was then processed to 3-oxoadipyl-CoA by upregulated 3-oxoadipate CoA transferase. At the final stage, the 3-Oxoadipyl -CoA was converted to succinyl-CoA and acetyl-CoA by 3-oxoadipyl-CoA thiolase (Fig. 7). In anaerobic degradation pathway, toluene was carboxylated to phenylacetate via phenyl acetate pathway as shown in Fig. 8.

### Chemotaxis

The complete genome sequencing elucidated that this strain is capable of high chemotactic ability, through a mechanism that involve flagellar protein for controlling the movement, which would be constructive for further development towards bioremediation approach (Supplementary File).

### Biosurfactant production

The genome sequencing revealed that this strain harbors genes encoding the biosynthesis of glycolipids associated with biosurfactant production, such as 3-oxoacyl-ACP reductase, acyltransferase, phosphomannomutase, and glycosyltransferase (Supplementary File), which are essential for initiating the biodegradation of complex hydrocarbon.

## Discussion

To the best of our knowledge, this is the first genome report on the piezotolerant *N*. *exalbescens* isolated from deep sea sediment and also the first study on toluene degradation at *in situ* deep sea conditions for possible bioremediation application in marine environment. Deep sea microorganisms have evolved multitude of physiological and genetic mechanisms to grow in adverse environmental conditions, such as extreme pH, salinity, pressure and temperature, and could have adapted to catalyze diverse functions. Many researchers have emphasized the importance of deep sea isolates towards broad tolerance to conditions such as pH, temperature, pressure and salinity^15,16^. Our previous work highlighted the unique phenotypic characterization and metabolic capability of consortium developed with different deep sea bacterial isolates^6^. The hydrocarbon degraders acclimatized to grow in variable conditions may play important role in degradation and removal of toxic pollutants existing in extremophilic environment. Ability of the culture to grow at variable temperature and salt concentration is noteworthy because many studies highlighted its importance on microbial degradation of hydrocarbon^17,18^. The closely related genera *Nesiotobacter exalbescens* (Fig. 1) is clearly differentiated from our isolate by the ability to grow on above 4% (w/v) NaCl^19^. The isolate assimilated aromatic hydrocarbons as their sole carbon source and grew on a variety of surfactant groups. Often bioremediation applications depend on enhancing hydrocarbon bioavailability by using surfactants. Interestingly, surfactant-resistant bacteria can be used in modification of affinity and adsorption properties of hydrophobic contaminants, and also can be used extensively for bioremediation of hydrocarbon pollution. Moreover, it can prevent the toxic effects of synthetic surfactants that often delay, reduce or prevent microbial bioremediation of pollutants^20^. This analysis confirmed that the deep sea isolate *N*. *exalbescens* COD22 posses surfactant-resistant property which is very important for bioremediation applications.

The selected 0.1 MPa and 10 MPa conditions were ideal for mineralization of all the petroleum hydrocarbons in marine environment. Interestingly, the growth pattern and biodegradation profile was dissimilar at contrasting pressure conditions. At 0.1 MPa, a short lag phase was followed by the exponential growth phase, whereas an extended lag phase was observed at 10 MPa condition. The high pressure system provoked an adaptive physiological and biochemical mechanism that induced intracellular changes required to acclimatize before proceeding to exponential growth phase. An extended lag phase in *Escherichia coli* at 60 MPa was observed and it was suggested that significant molecular dynamics in the cells were affected by changes in growth patterns associated with pressure variations^21^. The period of exponential growth was followed by a long stationary phase from 48 h, lasting up to 72 h. A distinct phase difference was observed at the stationary phase during which the cells were active for a longer period of time and cell density was higher at 0.1 MPa than 10 MPa. The *N*. *exalbescens* COD22 could grow with toluene, utilizing it as the sole carbon source under both low and high pressure conditions at the rate of 0.24 h^−1^ and 0.12 h^−1^, respectively. At 10 MPa growth condition, prolonged lag phase was required for intracellular adaptation to external changes thereby elucidated the relationship between growth rate and pressure. At 0.1 MPa pressure conditions, toluene biodegradation were high and the percentage of biodegradation was 50% at 24 h and 87.5% at 72 h. At 10 MPa pressure conditions, biodegradation was much more efficient and the percentage of biodegradation was 33%, 85% and 92% at 24 h, 48 h and 72 h, respectively (Fig. 2). The cell biomass in high-pressure reactor stabilized after 24 h of acclimatization period might be a factor for enhanced biodegradation. Qu *et al*., reported complete degradation of toluene by *Pseudomonas thivervalensis* at 6.59 mgL^−1^ day^−1^ ^22^. Significant biodegradation of benzene, toluene and xylene by *Pseudomonas* sp. was observed within a period of 21–42 h^23^. Physiological, biochemical and metabolic potentials along with adaptive physiological mechanisms of *N*. *exalbescens* COD22 might have driven positively for enhanced toluene degradation at two different pressure conditions. Microbial communities attempt to intertwine with all ecological parameters, although the complexity of deep sea marine ecosystems poses a vast set of challenges resulting in emergences of different survival mechanisms.

High pressure variation at different depths is one of the greatest challenges faced by microbial community for bioremediation application at marine habitat. Thus deep sea bacteria behavior may be different at *in situ* conditions compared to the growth and metabolic behavior at the laboratory conditions. Moreover, *in situ* biodegradation rates would be slow when compared to the moderate to warm temperature environment. Recent investigations have reported the breakdown of different hydrocarbons by diverse deep sea microbial groups, which includes alkane by *Desulfosarcina*/*Desulfococcus*^24^, polyaromatic hydrocarbons by *Pseudomonas mendocina*^25^ and crude oil by *Dietzia* sp.^26^. Interestingly, the deep sea isolate *Nesiotobacter* sp. can cope up with changing biotic conditions; thereby playing a vital role in the *in situ* bioremediation of oil spills. A report by Dashti *et al*., suggested that the genus *Nesiotobacter*, hydrocarbonoclastic bacteria is a potential source in the bioremediation of hydrocarbon contaminants^27^. The present study demonstrated the effect of piezotolerance on growth phase of *N*. *exalbescens* COD22. Based on our observations, we confirmed that two strategies were adopted by the cells in response to pressure changes. The cells aggregated in clusters to readily utilize the available nutrients from surrounding environment and counteracted the turbulence induced by external pressure variations (Fig. 3). To acclimatize to varying levels of hydrostatic pressure, the morphology of cells, adsorption and degradation rate of hydrocarbon would change accordingly^8^ and our results corroborates with these findings. Similarly, Jean *et al*., observed clustering of *Pseudomonas* sp. during benzene, toluene and xylene degradation in a sand aquifer model^23^. Careful observation of Kato and Qureshi elucidated the changes in the rate of respiration in response to change in the pressure^28^. This may be compared to the gene regulation in synthesis of fatty acids and proteins in piezophiles based on pressure^29^. These properties may be possible reason for the changes in the morphology in *N*. *exalbescens* COD22.

In FTIR, the major bands observed at 2853, 2925 and 2956 cm^−1^ in 0^th^ day have been assigned to νC–H of the aromatic ring and to asymmetric and symmetric νC–H of methyl group (Fig. 4a). In the period of 72 h, the disappearances of peaks were observed in the range of 1500–1600 cm^−1^, which may be due to the aromatic ring cleavage induced by microbial metabolism. Upon degradation, intensity of the peak at 1640 cm^−1^ began to decrease, meanwhile, the band at 1100 cm^−1^ corresponding to fermentation metabolites increased upon progress on biodegradation. The aromatic C–H stretching vibrations usually occurring between 3000–3100 cm^−1^ did not appear due to weaker C–H stretch in aromatics. Specifically, the spectrum was characterized by a sharp bands at 1740–1745 cm^−1^ corresponding to C=O stretching vibrations of carbonyl groups (Fig. 4b). The C–H stretch of aliphatic was found at 970 cm^−1^. The bands observed at 2955, 2928 and 2856 cm^−1^ can be assigned to both aromatic C–H and methyl C–H stretching vibrations. The band at 720 cm^−1^ is assigned to the vibrations of CH2 groups^30^. The carbonyl stretching of C=O aliphatic aldehydes increased in their intensity and appeared from 1680–1720 cm^−1^. The breakdown of aromatic compounds is indicated by the appearance of new band at 1715 cm^−1^ related to C=O carbonyl group. The absence of peaks at 3388.93, 3417.86, 3441.01, 2113.98, 2017.54, 1099.43, and 621.08 indicates the degradation of toluene (Fig. 4c). The results obtained by GC-MS analyses showed an extensive breakdown of toluene comprising metabolites, such as 3-ethoxy-4-methoxyphenol at *Rt* (47.284), 1,2-benzenedicarboxylic acid at *Rt* (55.622) and 1, 3-benzenediol at *Rt* (84.035) accumulating within 24 h at 10 MPa growth conditions. The *Rt* at 32.503 and 40.328 correspond to benzenepropanoic acid and benzoic acid (Table 2). The accumulation of carboxylic acid, benzyl alcohol and benzaldehyde has been reported previously as intermediates during aromatic compounds mineralization^31^. Many different pathways have been reported for aerobic toluene degradation. *Burkholderia fungorum* converts toluene by ortho cleavage enzymes and forms methylcyclohexa-3, 5-diene-1, 2-diol, 3-methylcatechol, 2-methyl-muconate and 2-methyl-2-enelactone as key intermediates^32^. *Ralstonia pickettii* transforms toluene to *p-*cresol and 4-methylcatechol^33^. The genome analysis correlates as well with the experimental results and identified the genes responsible for degradation and key chemical reactions involved in the pathway. *N*. *exalbescens* COD22 strain exhibited genes associated with utilization of aromatic compounds and motility. The main breakdown pathway of PAHs started by dioxygenases and cytochrome P450 monooxygenases. These enzymes are central to the degradation and conversion of breakdown metabolites. In addition to the main pathways, *Nesiotobacter* sp. possesses genes for caprolactam, biphenol and styrene degradation. In this context, it was clear that this hydrocarbonoclastic deep sea bacterium might possess still many pathways which may connect various intermediate reactions related to oil degradation network. The genome of *N*. *exalbescens* COD22 has specified genes that can involve in aerobic and anaerobic type of degradation. Also complete set of genes encoding motility, transport system and chemotaxis factors were present in the genome. The genes responsible in major biosynthetic pathways for carbohydrate metabolism, amino acid metabolism, membrane transport, metabolism of co-factors and vitamins, nucleotide metabolism, energy metabolism, signal transduction, transcription, translation, replication and repair, lipid metabolism, metabolism of terpenoids and polyketides, glucan biosynthesis and xenobiotics biodegradation were also identified (Fig. 6). This result concluded that the degradation of PAHs requires the coordinated response of varied genes for efficient utilization. The PAHs were transformed into key intermediate protocatechuate and metabolized completely. The genes protocatechuate 3,4-dioxygenase, alpha subunit and dioxygenase beta chain are related to β-ketoadipate pathway. Interestingly, sequencing showed PhaA, PhaB and enoyl-CoA hydratase/isomerase groups responsible for phenylacetate pathway for degradation. The presence of genes constituting aromatic compounds degradation include: Benzoate degradation (3-octaprenyl-4-hydroxybenzoate carboxylyase, acetyl-CoA C-acyltransferase activity, 3-oxoadipyl-CoA thiolase activity, p-hydroxybenzoate hydroxylase, 4-hydroxybenzoate octaprenyltransferase, 2-succinylbenzoate–CoA ligase); Phenol (Acetoin:2,6-dichlorophenolindophenol oxidoreductase, 2-octaprenyl-6-methoxyphenol hydroxylase) and aromatic ring cleavage (Extradiol ring-cleavage dioxygenase, class III enzyme, subunit B, Quercetin 2,3-dioxygenase, Gamma-butyrobetaine dioxygenase, nitronate monooxygenase). Multitude of enzymes belonging to PAHs degradation pathways were observed in *N*. *exalbescens* COD22. The complete genome analysis also revealed the presence of numerous oxidoreductases, hydroxylases, dehydrogenases, and dioxygenases related to the degradation of PAHs, cyclic hydrocarbons, and other aromatic compounds (Supplementary File). Laboratory analysis revealed the presence of aerobic and anaerobic metabolites of toluene. This observation is fully consistent with the enzymatic profile data and data on genome sequencing (Figs. 7 and 8). The genome sequence of strain *N*. *exalbescens* COD22 offers the possibility to investigate the basis of chemotaxis, for which the genes and/or pathways are still unknown. This strain has more than 10 genes for type VI pili assembly, better expression of pili genes could be a reason for mediating biofilm formation and adhesion to different surfaces^34^. The genome sequence revealed several specialized genes which code for chemotaxis and cell motility. Multiple homologues of bacterial chemotaxis genes including CheA, CheB, CheR and CheW were found in the genome. The response regulators CheY was activated by the cytoplasmic chemotaxis proteins, such as CheA, CheB, CheR and CheW^35^. This species also exhibits non-paralogous receptor CtpH and paralogous receptors PctB which mediate chemotaxis. The flagellar motor supramolecular complex includes the proteins FliE, FliG, FliK, FliL, FliM, FliN, FliO, FliP, FliQ and FliR. The proton channel was formed with MotB and MotC. The SwrC directly contributes to the swarming ability. Interestingly, most of the genes required for flagellar formation and production (FlgA, FlgB, FlgC, FlgE, FlgF, FlgG, FlgK, FlgL, FlhA, FlhB, FlbT, FtcR and FlaF) were found in clusters (Supplementary File). Hydrocarbonoclastic bacteria respond effectively to changes in the chemical constituents of their eco-system, move to relatively better niches and attach to the oil-water interface. This chemotactic behavior is dominantly achieved by incorporating the received signals and changing flagellar movement. Hydrocarbonoclastic microorganisms have unmatched capability to tolerate the toxicity of complex hydrocarbons by exhibiting appropriate emulsifying and degrading activities in the prevailing environments. Surface active biosurfactants promote emulsification and micellization by which the hydrophobic moieties are transferred into aqueous phase^36^. These properties solubilize hydrocarbons by lowering the interfacial surface tension facilitating its removal to overcome degradation problems. The genome sequencing revealed that this species harbors genes encoding the biosynthesis of glycolipids associated with biosurfactant production, such as 3-oxoacyl-ACP reductase, acyltransferase, phosphomannomutase, and glycosyltransferase (Supplementary File), which are essential for initiating the biodegradation of complex hydrocarbon. In this genome the lipid moiety of biosurfactant was prompted through the series of key genes including *accA*, *accB*, *accC*, *accD*, *fabA*, *fabB*, *fabD*, *fabF*, *fabA*, *fabG*, *fabH*, *fabI* and *fabZ* through the classical pathway of fatty acid synthesis^37^. Most of the genes involved in the biosynthesis of dTDP-L-rhamnose including *algA*, *algC* and *rmlD* were found in analysis (Supplementary File). The rhlG gene mediates 3-oxoacyl-[acyl-carrier-protein] reductase which was involved in rhamnolipid biosynthesis^38^. The tie-in interaction between biosurfactant and hydrocarbon biodegradation is of great interest which leads to application in microbial enhanced oil recovery.

## Methods

### Enrichment and isolation

The sediment from Bay of Bengal (13°14′192″N/80°49′061″E) was collected at 2100 m depth, using the research vessel Sagar Manjusha. Sub-samplings were carried out using sterile plastic syringe (50 mL) under sterile conditions and stored at 4 °C until usage. The enrichment was carried with 0.1% (v/v) toluene on mineral salt medium (MSM) containing NaCl (1%), KCl (0.1%), KH_2_PO_4_ (1%), K_2_HPO_4_ (1.5%), (NH_4_)_2_SO_4_ (0.75%), MgSO_4_.7H_2_O (0.3%) and peptone (0.1%). Following several purification steps, toluene degrader was isolated from enrichment medium. Isolated toluene degrader was purified to monoculture and stocked.

### Biochemical and molecular characterization

The bacterium was grown in Zobell marine broth (HiMedia, India) using sterilized sea water and incubated at 28 °C. The biochemical characterizations were done by using KB002, KB009A, KB009B and KB009C (HiMedia, India) based on standard procedures. The bacterial culture (50 µl) grown at log phase was transferred onto each well in the test kits and incubated at 37 °C for 24 h. The results were checked after appropriate incubation by comparing to biochemical test result chart accordingly to manufacturer instruction. Molecular characterization included extraction of genomic DNA followed by amplification using 16S rRNA primer (27F AGAGTTTGATCCTGGCTCAG and 1492R GGTTACCTTGTTACGACTT) and sequenced. The obtained sequences were aligned in MEGA6 and phylogenetic tree was constructed using neighbour-joining method. The genomic DNA G + C% was measured using thermal denaturation method.

### Bacterial growth physiology

The growth of culture at differential concentration of NaCl (0–10% w/v) was investigated on marine broth (peptone 0.05 g/L, yeast extract 0.01 g/L, ferric citrate 0.001 g/L, MgCl_2_.6H_2_O 0.059 g/L, MgSO_4_.7H_2_O 0.032 g/L, CaCl_2_.2H_2_O 0.018 g/L, KCl 0.006 g/L, KBr 0.0008 g/L and H_3_BO_3_ 0.0002 g/L). The temperature required for optimal growth was tested at 10, 20, 28, 37 and 45 °C. The growth pH was tested at a range of 5.0 to 10.0 pH.

### Substrate specificity

Substrates (0.1%) such as brij-35, cedar wood oil, crude oil, diesel, hexadecane, kerosene, petrol, sodium dodecyl sulphate, spent engine oil, toluene, triton X-100, tween 80, xylene, silicone oil, phenol and clove oil were added onto mineral salt medium and inoculated with the test microorganism. The utilization of carbon source was monitored by measuring increase in cell density. The test culture was incubated at 28 °C for 120 h and cell growth was monitored every 24 h.

### Deep-sea microbial culture facility (DMCF)

**Figure 9:**
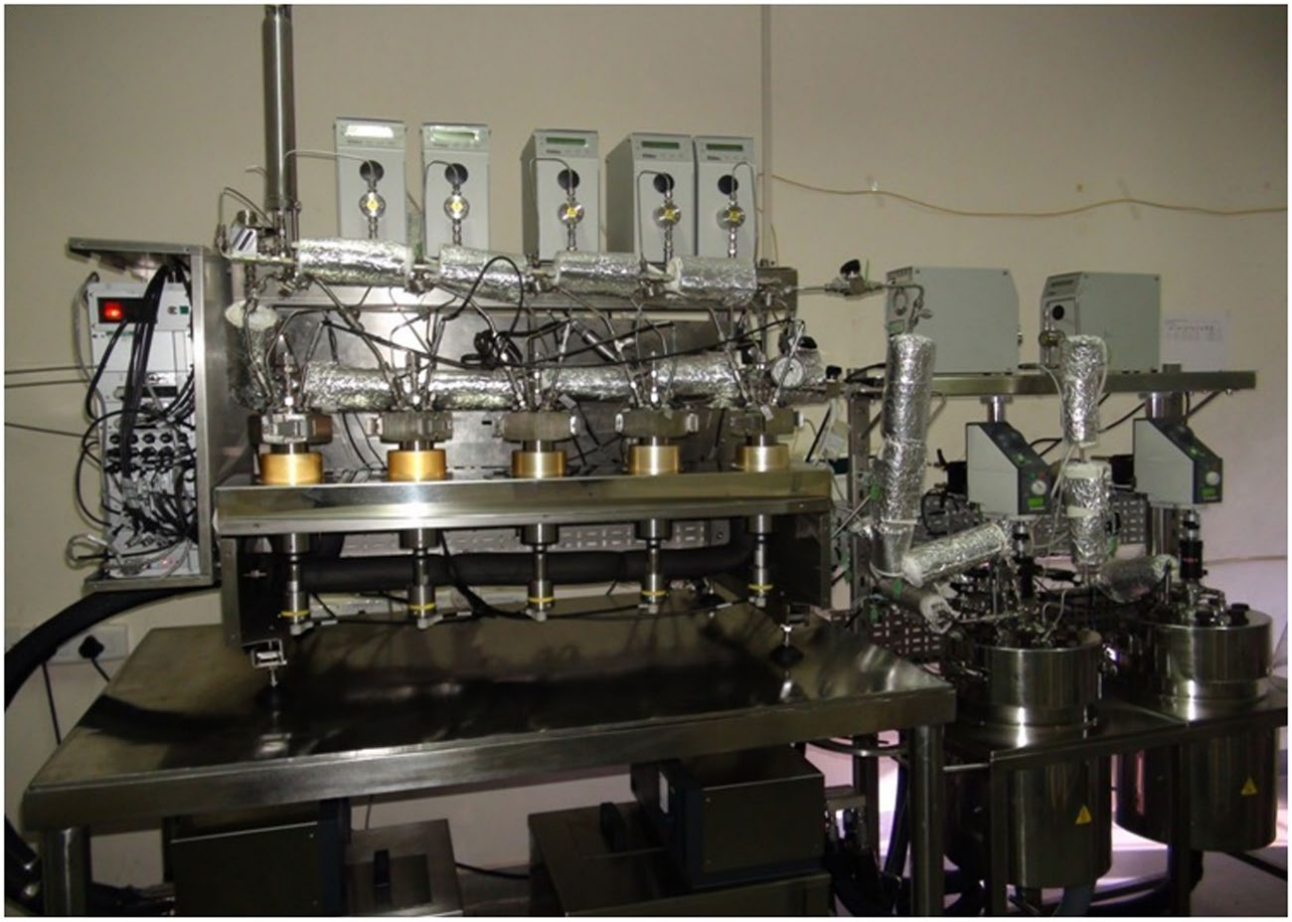
Deep-sea microbial culture facility (DMCF) reactor used for culturing *N*. *exalbescens* COD22 at 10 MPa pressure conditions.

DMCF facilitates isolation and cultivation of microbes at *in-situ* deep sea conditions during the entire process without any major change in the environmental parameters such as pressure, temperature, pH and Dissolved Oxygen. The maximum operating pressure of DMCF was 35 MPa. The serial dilution system (SDS) has five numbers of 100 mL reactor vessels. Two customized high pressure stainless steel reactors (ESSO-NIOT, India) with working volume of 5 L were integrated with SDS to investigate biodegradation of toluene under elevated pressure conditions (Fig. 9). The complete culture facility was 35 MPa rated and by using these modules we can dilute the microbes to any fold and cultivate without any major change in the *in-situ* conditions. The DMCF have the provision to sterilize up to 225 °C and this makes easy to carryout *in-situ* biological experiments without external contamination.

### Hydrocarbon biodegradation

The pressure tolerance of *Nesiotobacter* sp. was studied in MSM prepared in sterilized sea water and supplemented with glucose (0.1% w/v). After sterilization, trace salt solution (1%) comprising of CaCl_2_ (20.0 mg), FeCl_3_ (30.0 mg), CuSO_4_ (0.5 mg), MnSO_4_.H_2_O (0.5 mg), and ZnSO_4_.7H_2_O (10.0 mg) per liter was filter sterilized and added to the medium. The log phase culture (2%) was inoculated into the culture vessels. These cultures were grown at 0.1 and 10 MPa (1 and 100 bar) pressure in 28 °C and 20 °C, respectively, for 120 h. Samples were collected in duplicates at every 24 h and analyzed. Same methodology was followed for biodegradation analysis where toluene (2 mM) was added to the MSM instead of glucose. Polysorbate 80, non-ionic surfactant 0.001% (v/v) was added to all tests to provide uniform emulsification of toluene. The high pressure culture vessels were pre-sterilized and pressurized using filtered nitrogen gas to a maximum level of 10 MPa. In order to overcome the pressure induced damage in the bacteria, the pressure was raised gradually from 0.1 MPa to 10 MPa for 24 h, after which the pressure was maintained constant. Bacterial growth (OD at 600 nm and colony forming units-CFU/mL) and toluene percentage were analyzed to measure the degradation.

### Bacterial growth measurement

The growth of the bacteria was assessed by measuring absorbance at 600 nm (A600 nm) with a Unicam UV 300 spectroscopy (Thermo Spectronic). Colony forming units (CFU) was determined by plating of marine agar plates in duplicates. After 48 h of incubation at room temperature, total colonies were counted.

### Morphological analysis-scanning electron microscopy

The morphological changes at ambient and elevated pressure condition were studied by SEM. Bacterial cells were sampled at different times during the exponential phase of growth, fixed with 2.5% (v/v) glutaraldehyde for 60 min. The fixed cells were dehydrated in an increasing gradient of ethanol concentration up to 100%. This was continued by CPD drying with CO_2_, sputtered with gold (BAL-TEC SDC) and visualized in SEM (TESCAN, vega 3, USA).

### Analytical methods - determination of toluene degradation

Toluene was quantified by extracting the percentage of unused toluene in 100 mL sample. In brief, the sample was extracted with 40 mL of n-hexane and chloroform (2:1) mixture two times and solvent phase was separated. The organic phase was analyzed in UV spectroscopy. The readings were taken every 24 h up to 120 h and the estimation was done as the mean of three independent measurements.

### FTIR and GC-MS analysis

Toluene degradation at two different physiological conditions was characterized by FTIR spectroscopy (IR affinity-1 Shimadzu spectrophotometer, Japan) in the range 4000 to 400 cm^−1^. The breakdown products of toluene at different time intervals were recorded by analysis in GC-MS. After solvent extraction, the organic phase was concentrated completely under stream of nitrogen and during sample analysis it was reconstituted in n-hexane. The GC-MS analysis was carried out using, AGILENT (GC 7890 A, USA) mass spectrometry under external ionization mode with a column HP-5 MS (30 m × 0.32 mm × 0.25 µm) with helium as the carrier gas.

### Library preparation and genome sequencing

Genomic DNA was isolated from 10 mL of culture using QIAamp DNA mini kit (Qiagen, Valencia, USA) as per the protocol given by the manufacturer. The quality and purity of the genomic DNA was checked using agarose gel electrophoresis and Qubit 3 Fluorometer, respectively. Based on agarose gel electrophoresis, the quantity was verified using the Qubit dsDNA HS assay kit for precise measurements. The whole genome was sequenced by using paired-end sequencing (265 × 2) with a Miseq sequencing system (Illumina). The raw data obtained was processed to remove the adapters and the low quality bases with Q-score less than 20. The pre-processed quality reads was used for the *de novo* genome assembly. SPAdes assembler (version v3) was used for *de novo* assembly after error-correction of sequenced reads. The bacterial chromosome map was generated using BRIG-v0.95 tool. Gene prediction was done using Prodigal. Similarity search for the predicted genes were done against Uniprot α-Proteobacteria. Protein database for Gene Ontology (GO) was done using DIAMOND program and pathway analysis was carried out using KAAS Server.

## Conclusion

*N*. *exalbescens* COD22 is a novel deep sea strain and degraded 87.5 and 92% of toluene at ambient and high pressure growth conditions within a short incubation period of 72 h. Toluene degradation pathway has been elucidated for the first time in deep sea strain under high pressure conditions. Within 24 h of incubation, benzene was converted to phenol, as evident by the formation of lactotoluene. Overall, the results provided substantial evidence that toluene is degraded and utilized by *Nesiotobacter* sp. via a combined pathway which is not analogous to that found in previously described toluene degraders^39^. Furthermore, the results indicated that the formation of phenol was due to enzymatic hydroxylation of benzene by specific hydroxylases irrespective of elevated pressurized conditions. The present study showed a distinct growth pattern and degradation of toluene that occurred significantly faster than biomass accumulation at elevated pressure conditions. These results provided conclusive evidence for the ability of *N*. *exalbescens* COD22 for possible bioremediation applications in marine ecosystem. The genome analysis elucidated the degradation pathways for complex aromatic compounds and underlying mechanism for chemotaxis, biosurfactant synthesis, pili and well developed flagellar assembly which can function as adaptive responses to emulsify the hydrocarbons for degradation of crude oil. These findings on biodegradation at high pressure growth conditions and whole genome sequencing analyses provide mastery insight for designing remediation strategy for the restoration of hydrocarbon polluted deep sea environments.

## Supplementary information

Supplementary file
