# Publisher Correction: Genome analysis of deep sea piezotolerant *Nesiotobacter exalbescens* COD22 and toluene degradation studies under high pressure condition

**DOI:** 10.1038/s41598-020-75726-x

**Published:** 2020-10-28

**Authors:** A. Ganesh Kumar, Noelin Chinnu Mathew, K. Sujitha, R. Kirubagaran, G. Dharani

**Affiliations:** Marine Biotechnology Division, Earth System Science Organization - National Institute of Ocean Technology (ESSO – NIOT), Ministry of Earth Sciences (MoES), Government of India, Pallikaranai, Chennai, 600100 India

Correction to:* Scientific Reports* 10.1038/s41598-019-55115-9, published online 10 December 2019

In the original version of this Article, the authors were incorrectly indexed. This error has now been corrected.

