# Supplementary material for: Genome analysis of deep sea piezotolerant Nesiotobacter exalbescens COD22 and toluene degradation studies under high pressure condition
Source: Sci Rep. 2019 Dec 10;9:18724. doi: 10.1038/s41598-019-55115-9 (PMC6904484; doi:10.1038/s41598-019-55115-9)
Supplement: Supplementary file 1 — Supplementary file [file 41598_2019_55115_MOESM1_ESM.pdf]

Genome analysis of deep sea piezotolerant *Nesiotobacter exalbescens* COD22 and toluene degradation studies under high pressure condition

A. Ganesh Kumar*, Noelin Chinnu Mathew, K. Sujitha, R. Kirubagaran and G. Dharani

Marine Biotechnology Division, Earth System Science Organization - National Institute of Ocean Technology (ESSO – NIOT), Ministry of Earth Sciences (MoES), Government of India, Pallikaranai, Chennai-600100, India

Supplementary file: Polycyclic aromatic hydrocarbon degrading genes

**Polycyclic aromatic hydrocarbon degrading genes**				
**Protein ID(Subject ID)**	**% Identity**	**Protein Names**	**Gene name**	**Gene Ontology IDs**
tr|G8PKK8|G8PKK8_PSEUV	81.8	Protocatechuate 3,4-dioxygenase beta chain (EC 1.13.11.3)	pcaH PSE_2279	GO:0006725 GO:0008199 GO:0018578 GO:0019619
tr|B6R3H6|B6R3H6_9RHOB	73.4	Protocatechuate 3,4-dioxygenase, alpha subunit (EC 1.13.11.3)	pcaG PJE062_637	GO:0008199 GO:0018578 GO:0019439
tr|A9CIX2|A9CIX2_AGRFC	38.7	Cytochrome P450	cyc Atu1569	GO:0004497 GO:0005506 GO:0016705 GO:0020037
tr|A0A165UL10|A0A165UL10_9RHOB	79.6	3-oxoacyl-[acyl-carrier-protein] reductase FabG (EC 1.1.1.100)	fabG_5 PsAD2_03796	GO:0004316 GO:0006633 GO:0051287 GO:0102132
tr|A0A161UB03|A0A161UB03_9RHOB	68.9	p-hydroxybenzoate hydroxylase (EC 1.14.13.2)	pobA PsW74_02134	GO:0018659 GO:0043639 GO:0071949
tr|G8PSH8|G8PSH8_PSEUV	75.6	Homoserine kinase (HK) (HSK) (EC 2.7.1.39)	thrB PSE_1572	GO:0004413 GO:0005524 GO:0009088
tr|A0A165Z4N9|A0A165Z4N9_9RHOB	89	Acyl-CoA dehydrogenase (EC 1.3.99.-)	mmgC_1 PsAD2_01784	GO:0003995 GO:0050660

tr|A0A165N7Y4|A0A165N7Y4_9RHOB	70.3	Thiol:disulfide interchange protein CycY	cycY PsW74_05070	GO:0015036 GO:0016021 GO:0017004 GO:0030288 GO:0045454
tr|G8PKL2|G8PKL2_PSEUV	74.9	Beta-ketoadipyl-CoA thiolase (EC 2.3.1.16)	pcaF PSE_2283	GO:0003988 GO:0019619
tr|A0A166DR12|A0A166DR12_9RHOB	90.5	Muconate cycloisomerase 1 (EC 5.5.1.1)	catB PsWM33_01822	GO:0008152 GO:0018849 GO:0046872
tr|G8PSZ0|G8PSZ0_PSEUV	62.5	Mandelate racemase/muconate lactonizing enzyme family protein	PSE_4128	GO:0003824 GO:0008152 GO:0016021 GO:0046872
tr|A0A196QD58|A0A196QD58_9RHOB	47.3	3-carboxy-cis,cis-muconate cycloisomerase	A8B74_03560	GO:0016853
tr|A0A1A5S1Z0|A0A1A5S1Z0_9RHIZ	49.6	3-oxoadipate enol-lactonase	A9K71_19240	GO:0042952 GO:0047570
tr|H0HJJ0|H0HJJ0_9RHIZ	75	4-carboxymuconolactone decarboxylase	MAXJ12_01476	GO:0051920
tr|A0A165SCA9|A0A165SCA9_9RHOB	86.1	Ubiquinone/menaquinone biosynthesis C-methyltransferase UbiE (EC 2.1.1.163) (EC 2.1.1.201) (2-methoxy-6-polyprenyl-1,4-benzoquinol methylase) (Demethylmenaquinone methyltransferase)	ubiE_1 ubiE PsW74_00681	GO:0006744 GO:0008425 GO:0009060 GO:0009234 GO:0102005
tr|G8PKK6|G8PKK6_PSEUV	76.2	HTH-type transcriptional regulator pcaQ (Pca operon transcriptional activator)	pcaQ PSE_2277	GO:0003677 GO:0003700 GO:0006351 GO:0019619 GO:0045893
tr|B6R1N2|B6R1N2_9RHOB	75.9	3-octaprenyl-4-hydroxybenzoate carboxy-lyase	PJE062_2675	GO:0010181 GO:0016491 GO:0016831
tr|G8PKL2|G8PKL2_PSEUV	74.9	Beta-ketoadipyl-CoA thiolase (EC 2.3.1.16)	pcaF PSE_2283	GO:0003988 GO:0019619
tr|G8PFI5|G8PFI5_PSEUV	73.8	Ubiquinone biosynthesis O-	ubiG PSE_0011	GO:0006744 GO:0008425

		methyltransferase (2-polyprenyl-6-hydroxyphenol methylase) (3-demethylubiquinone 3-O-methyltransferase)		GO:0061542
tr|A0A166AFL4|A0A166AFL4_9RHOB	70.5	Succinyl-CoA:(R)-benzylsuccinate CoA-transferase subunit BbsF (EC 2.8.3.15)	bbsF_2 PsAD2_00995	GO:0033877
tr|A0A0M6ZHP8|A0A0M6ZHP8_9RHOB	63.9	4-hydroxybenzoate octaprenyltransferase (EC 2.5.1.-) (4-HB polyprenyltransferase)	ubiA LA5096_03316	GO:0005886 GO:0006744 GO:0008412 GO:0016021
tr|A0A166BH97|A0A166BH97_9RHOB	56.2	p-hydroxybenzoate hydroxylase transcriptional activator	pobR PsWM33_04028	GO:0003677 GO:0006351 GO:0006355
tr|A0A0B4XYZ1|A0A0B4XYZ1_9PROT	47.1	Benzoate transporter	TH3_07035	GO:0016021 GO:0042925
tr|B6R0L7|B6R0L7_9RHOB	49	Salicylate 1-monooxygenase	PJE062_4552	GO:0004497 GO:0016021 GO:0071949
tr|A0A165U4S0|A0A165U4S0_9RHOB	58.2	2-succinylbenzoate--CoA ligase (EC 6.2.1.26)	menE PsAD26_03203	GO:0008152 GO:0008756
tr|A0A166EXP5|A0A166EXP5_9RHOB	91.8	Anaerobic benzoate catabolism transcriptional regulator	PsWM33_00597	GO:0043565
**Biphenol degrading genes**				
tr|B6R5P0|B6R5P0_9RHOB	64.2	Cupin superfamily protein	PJE062_3257	NA
**Caprolactam degrading genes**				
tr|A0A165Z4W9|A0A165Z4W9_9RHOB	78.1	Putative enoyl-CoA hydratase echA8 (EC 4.2.1.17)	echA8_1 PsAD2_01790	GO:0004300 GO:0008152
tr|B6R9C2|B6R9C2_9RHOB	73.9	Enoyl-CoA hydratase/isomerase	PJE062_4050	GO:0008152 GO:0016853
GO:0004300; GO:0008152	69	Enoyl-CoA hydratase/isomerase	NA	GO:0008152 GO:0016853

tr|F2IYW5|F2IYW5_POLGS	66.3	Enoyl-CoA hydratase / short chain enoyl-CoA hydratase	SL003B_2155	GO:0003824 GO:0010124
tr|A0A165XZ19|A0A165XZ19_9RHOB	62.1	Putative enoyl-CoA hydratase 1 (EC 4.2.1.17)	PsAD2_02651	GO:0004300
tr|A0A165S531|A0A165S531_9RHOB	58.7	Putative enoyl-CoA hydratase echA8 (EC 4.2.1.17)	echA8_4 PsW74_01117	GO:0003860 GO:0004300
tr|A0A165VGP1|A0A165VGP1_9RHOB	52.9	2,3-dehydroadipyl-CoA hydratase (EC 4.2.1.17)	paaF_1 PsAD26_01621	GO:0004300 GO:0008152
tr|C3MH79|C3MH79_SINFN	51.2	Enoyl-CoA hydratase/isomerase/3-hydroxyacyl-CoA dehydrogenase	NGR_c26080	GO:0003857 GO:0006635 GO:0016853
tr|B6R524|B6R524_9RHOB	72.8	3-hydroxyacyl-CoA dehydrogenase (EC 1.1.1.35)	fadB PJE062_78	GO:0003857
**Chloro-hexane and benzene degrading genes**				
tr|K0PCN2|K0PCN2_9RHIZ	69.7	Predicted dienelactone hydrolase	BN77_1474	GO:0003847 GO:0016042
tr|A0A0D6B8B4|A0A0D6B8B4_RHOSU	48	Dienelactone hydrolase	NHU_04228	GO:0016021 GO:0016787
tr|A0A166DR12|A0A166DR12_9RHOB	90.5	Muconate cycloisomerase 1 (EC 5.5.1.1)	catB PsWM33_01822	GO:0008152 GO:0018849 GO:0046872
tr|G8PSZ0|G8PSZ0_PSEUV	62.5	Mandelate racemase/muconate lactonizing enzyme family protein	PSE_4128	GO:0003824 GO:0008152 GO:0016021 GO:0046872
tr|A0A196QD58|A0A196QD58_9RHOB	47.3	3-carboxy-cis,cis-muconate cycloisomerase	A8B74_03560	GO:0016853
tr|A0A0M7A2C6|A0A0M7A2C6_9RHOB	69.1	(S)-2-haloacid dehalogenase 4A (EC 3.8.1.2)	hdl IVa LAX5112_01549	GO:0008152 GO:0018784
tr|A0A165WQ27|A0A165WQ27_9RHOB	45.2	Haloalkane dehalogenase (EC 3.8.1.5)	dhmA PsAD2_03399	GO:0018786
**Styrene degrading genes**				

tr|A0A0R3KL40|A0A0R3KL40_9BRAD	68.7	XRE family transcriptional regulator	CP49_18670	GO:0043565
tr|A1B1R0|A1B1R0_PARDP	66.9	Transcriptional regulator, XRE family	Pden_1350	GO:0006355 GO:0043565
tr|A0A0M6YQ95|A0A0M6YQ95_9RHOB	81.2	Putative succinyl-CoA:3-ketoacid coenzyme A transferase subunit B (EC 2.8.3.5)	scoB LP7551_01059	GO:0008152 GO:0008260
tr|G8PQV9|G8PQV9_PSEUV	91.3	GMP synthase [glutamine-hydrolyzing] (EC 6.3.5.2) (GMP synthetase) (Glutamine amidotransferase)	guaA PSE_2697	GO:0003922 GO:0005524 GO:0006177 GO:0006541 GO:0016462
tr|G8PNG7|G8PNG7_PSEUV	90.5	Aspartyl/glutamyl-tRNA(Asn/Gln) amidotransferase subunit C (Asp/Glu-ADT subunit C) (EC 6.3.5.-)	gatC PSE_3768	GO:0005524 GO:0006412 GO:0006450 GO:0016740 GO:0050567
tr|B6RA65|B6RA65_9RHOB	88.5	Aspartyl/glutamyl-tRNA(Asn/Gln) amidotransferase subunit B (Asp/Glu-ADT subunit B) (EC 6.3.5.-)	gatB PJE062_3516	GO:0005524 GO:0006412 GO:0016740 GO:0050567
tr|B6R0G4|B6R0G4_9RHOB	87.3	Phosphoribosylformylglycinamidine synthase subunit PurS (FGAM synthase) (EC 6.3.5.3) (Formylglycinamide ribonucleotide amidotransferase subunit III) (Phosphoribosylformylglycinamidine synthase subunit III)	purS PJE062_5093	GO:0004642 GO:0005524 GO:0005737 GO:0006189
tr|A0A165TVT5|A0A165TVT5_9RHOB	86.8	Glutamyl-tRNA(Gln) amidotransferase subunit A (Glu-ADT subunit A) (EC 6.3.5.7)	gatA_1 gatA PsAD2_04206	GO:0005524 GO:0006412 GO:0016740 GO:0050567

tr|B6R0G3|B6R0G3_9RHOB	86.5	Phosphoribosylformylglycinamidine synthase subunit PurQ (FGAM synthase) (EC 6.3.5.3) (Formylglycinamide ribonucleotide amidotransferase subunit I) (Glutaminase PurQ) (Phosphoribosylformylglycinamidine synthase subunit I)	purQ PJE062_4836	GO:0004642 GO:0005524 GO:0005737 GO:0006189 GO:0006541 GO:0016787
tr|G8PQX1|G8PQX1_PSEUV	83.5	Amidophosphoribosyltransferase (ATase) (EC 2.4.2.14) (Glutamine phosphoribosylpyrophosphate amidotransferase)	purF PSE_2709	GO:0000287 GO:0004044 GO:0006189 GO:0006541 GO:0009113 GO:0009116
tr|G8PS18|G8PS18_PSEUV	82.5	Phosphoribosylformylglycinamidine synthase subunit PurL (FGAM synthase) (EC 6.3.5.3) (Formylglycinamide ribonucleotide amidotransferase subunit II) (Glutamine amidotransferase PurL) (Phosphoribosylformylglycinamidine synthase subunit II)	purL PSE_4049	GO:0000287 GO:0004642 GO:0005524 GO:0005737 GO:0006189
tr|G8PM28|G8PM28_PSEUV	77.9	Imidazole glycerol phosphate synthase subunit HisH (EC 2.4.2.-) (IGP synthase glutamine amidotransferase subunit) (IGP synthase subunit HisH) (ImGP synthase subunit HisH)	hisH PSE_0558	GO:0000105 GO:0000107 GO:0005737 GO:0006541
tr|A0A165UKH3|A0A165UKH3_9RHOB	72.5	Glutamine--fructose-6-phosphate aminotransferase [isomerizing] (EC 2.6.1.16) (D-fructose-6-phosphate amidotransferase)	nodM glmS PsAD26_02541	GO:0004360 GO:0005737 GO:0005975 GO:0006541 GO:0030246 GO:1901137

		(GFAT) (Glucosamine-6-phosphate synthase) (Hexosephosphate aminotransferase) (L-glutamine--D-fructose-6-phosphate amidotransferase)		
tr|A9FUM4|A9FUM4_PHAIB	82.6	Aldehyde dehydrogenase (EC 1.2.1.-) (Succinate-semialdehyde dehdyrogenase GabD) (EC 1.2.1.16)	gabD3 PGA1_c23350 PGA1_c27190	GO:0009013
tr|A0A165Z5C8|A0A165Z5C8_9RHOB	76.7	NAD/NADP-dependent betaine aldehyde dehydrogenase (EC 1.2.1.8)	betB PsAD2_01802	GO:0008802 GO:0019285 GO:0046872
tr|G8PTQ8|G8PTQ8_PSEUV	90.7	Urease subunit alpha (EC 3.5.1.5) (Urea amidohydrolase subunit alpha)	ureC PSE_2971	GO:0005737 GO:0009039 GO:0016151 GO:0043419
tr|A0A165VT36|A0A165VT36_9RHOB	56.4	Maleylpyruvate isomerase (EC 5.2.1.4)	nagL PsAD2_03660	GO:0005737 GO:0009072 GO:0050077
tr|A0A0N0JST9|A0A0N0JST9_9PROT	37.9	Maleylacetoacetate isomerase	IP88_13250	GO:0005737 GO:0009072 GO:0016853
tr|B6YYZ3|B6YYZ3_9RHOB	75.5	Fumarylacetoacetase (EC 3.7.1.2)	fahA PJE062_3811	GO:0004334 GO:0009072
**Toluene degrading genes**				
tr|G8PW03|G8PW03_PSEUV	78.3	Monooxygenase FAD-binding protein	PSE_p0359	GO:0004497 GO:0071949
tr|A0A166DR12|A0A166DR12_9RHOB	90.5	Muconate cycloisomerase 1 (EC 5.5.1.1)	catB PsWM33_01822	GO:0008152 GO:0018849 GO:0046872
tr|A0A166AFL4|A0A166AFL4_9RHOB	70.5	Succinyl-CoA:(R)-benzylsuccinate CoA-transferase subunit BbsF (EC 2.8.3.15)	bbsF_2 PsAD2_00995	GO:0033877
tr|A0A161UB03|A0A161UB03_9RHOB	68.9	p-hydroxybenzoate hydroxylase (EC 1.14.13.2)	pobA PsW74_02134	GO:0018659 GO:0043639 GO:0071949

tr|A0A165XL31|A0A165XL31_9RHOB	57	Toluene efflux pump periplasmic linker protein TtgD	ttgD PsAD2_02925	GO:0016020 GO:0055085
tr|A0A0D6B8B4|A0A0D6B8B4_RHOSU	48	Dienelactone hydrolase	NHU_04228	GO:0016021 GO:0016787
**Napthalene degrading genes**				
tr|G8PPS4|G8PPS4_PSEUV	89.1	Aldehyde-alcohol dehydrogenase	adhE PSE_1331	GO:0004022 GO:0006066 GO:0008774 GO:0015976 GO:0046872
tr|G8PLH2|G8PLH2_PSEUV	90.3	Iron-containing alcohol dehydrogenase	PSE_2354	GO:0016491 GO:0046872
tr|B6R0L7|B6R0L7_9RHOB	49	Salicylate 1-monooxygenase	PJE062_4552	GO:0004497 GO:0016021 GO:0071949
**Butanoate degrading genes**				
tr|B6R246|B6R246_9RHOB	91.9	Succinate dehydrogenase iron-sulfur subunit (EC 1.3.5.1)	sdhB PJE062_2569	GO:0006099 GO:0008177 GO:0009055 GO:0046872 GO:0051537 GO:0051538 GO:0051539
tr|G8PLH2|G8PLH2_PSEUV	90.3	Iron-containing alcohol dehydrogenase	PSE_2354	GO:0016491 GO:0046872
tr|G8PIJ4|G8PIJ4_PSEUV	80.9	3-hydroxybutyryl-CoA dehydrogenase (EC 1.1.1.157)	hbd PSE_0271	GO:0003857 GO:0006631 GO:0008691 GO:0070403
tr|A9FUM4|A9FUM4_PHAIB	82.6	Aldehyde dehydrogenase (EC 1.2.1.-) (Succinate-semialdehyde dehdyrogenase GabD) (EC 1.2.1.16)	gabD3 PGA1_c23350 PGA1_c27190	GO:0009013

tr|A0A0M6YQ95|A0A0M6YQ95_9RHOB	81.2	Putative succinyl-CoA:3-ketoacid coenzyme A transferase subunit B (EC 2.8.3.5)	scoB LP7551_01059	GO:0008152 GO:0008260
tr|A0A0M2R695|A0A0M2R695_9PROT	70.1	Succinate-semialdehyde dehydrogenase	WH95_17675	GO:0016620
tr|Q216X6|Q216X6_RHOPB	56.2	Butyryl-CoA:acetate CoA transferase (EC 2.8.3.8)	RPC_2004	GO:0008152 GO:0008775
tr|A0A165VGP1|A0A165VGP1_9RHOB	52.9	2,3-dehydroadipyl-CoA hydratase (EC 4.2.1.17)	paaF_1 PsAD26_01621	GO:0004300 GO:0008152

Supplementary file: Chemotaxis protein

Chemotaxis protein				
**Protein ID (Subject ID)**	**% Identity**	**Protein Names**	**Gene name**	**Gene Ontology IDs**
tr|A0A165VCT3|A0A165VCT3_9RHOB	96.1	Chemotaxis protein CheY	cheY_1 PsAD26_01469	GO:0000160 GO:0005622
tr|F2IXP8|F2IXP8_POLGS	87.6	Response regulator receiver domain protein (CheY-like)	cheY SL003B_0950	GO:0000160 GO:0005622
tr|G8PJ68|G8PJ68_PSEUV	79.9	Chemotaxis protein CheW	cheW PSE_4652	GO:0004871 GO:0006935
tr|G8PJ69|G8PJ69_PSEUV	77.2	Chemotaxis protein CheA	cheA PSE_4653	GO:0000155 GO:0005737 GO:0006935
tr|A0A0L0J272|A0A0L0J272_9RHOB	71.2	Chemotaxis protein CheR	APZ00_09705	GO:0008757
tr|A0A165NGP8|A0A165NGP8_9RHOB	56.6	Chemotaxis regulator CheZ	PsW74_04921	GO:0003824 GO:0009288 GO:0050920
tr|A0A165PTB5|A0A165PTB5_9RHOB	65.1	Chemotaxis response regulator protein-glutamate methylesterase (EC 3.1.1.61)	cheB PsAD26_05255	GO:0000156 GO:0005737 GO:0006935 GO:0008984
tr|B6R898|B6R898_9RHOB	62.2	Chemotaxis protein	PJE062_5208	GO:0003824 GO:0009288 GO:0050920
tr|A0A165UFQ7|A0A165UFQ7_9RHOB	44.1	Chemotaxis protein LafU	lafU PsAD26_02650	
**Chemotaxis motility protein**				
tr|G8PKZ0|G8PKZ0_PSEUV	74.3	Chemotaxis protein MotB	motB PSE_4798	GO:0016021
tr|G8PPZ7|G8PPZ7_PSEUV	54.2	Chemotaxis protein MotC	motC PSE_2618	GO:0016020
tr|A0A165TYE4|A0A165TYE4_9RHOB	86.1	Motility protein A	motA PsAD2_04135	GO:0008565 GO:0016021
tr|F2IWH3|F2IWH3_POLGS	77.2	MotA/TolQ/ExbB proton channel	SL003B_0842	GO:0008565 GO:0016021
tr|A0A165R837|A0A165R837_9RHOB	56.3	Methyl-accepting chemotaxis protein McpB	mcpB PsW74_022	GO:0004871 GO:0006935

			74	GO:0016021
tr|A0A165RWX2|A0A165RWX2_9RHOB	54.1	Methyl-accepting chemotaxis protein PctB	pctB_2 PsW74_01706	GO:0004871 GO:0006935 GO:0016021
tr|A0A165RBX1|A0A165RBX1_9RHOB	52.8	Methyl-accepting chemotaxis protein 4	mcp4_6 PsW74_01937	GO:0004871 GO:0016020
tr|G8PQV3|G8PQV3_PSEUV	52.8	Methyl-accepting chemotaxis receptor/sensory transducer	PSE_2691	GO:0004871 GO:0006935 GO:0016021
tr|A0A166BQ02|A0A166BQ02_9RHOB	50.2	Heme-based aerotactic transducer HemAT	hemAT_2 PsWM33_03864	GO:0006935
tr|A0A165SQG1|A0A165SQG1_9RHOB	43.2	Methyl-accepting chemotaxis protein CtpH	ctpH_1 PsW74_00434	GO:0004871 GO:0006935 GO:0016021
tr|A0A0M6Z3V3|A0A0M6Z3V3_9RHOB	36.8	Methyl-accepting chemotaxis protein 2	mcp2_3 LP7551_05351	GO:0004871 GO:0006935 GO:0016021
**Flagella-driven chemotaxis**				
tr|A0A165TYD1|A0A165TYD1_9RHOB	97.8	Flagellar motor switch protein FliN	fliN_3 PsAD2_04132	GO:0003774 GO:0005886 GO:0006935 GO:0009425 GO:0071973
tr|G8PS56|G8PS56_PSEUV	85.6	Flagellar motor switch protein fliM	fliM PSE_4087	GO:0003774 GO:0009425 GO:0071973
tr|A0A166F2M6|A0A166F2M6_9RHOB	82.7	Flagellar basal-body rod protein FlgC	flgC_1 PsWM33_00457	GO:0030694 GO:0071973
tr|A0A165XT10|A0A165XT10_9RHOB	80.6	Flagellar motor switch protein FliG	fliG_1 PsAD2_02744	GO:0003774 GO:0005886 GO:0006935 GO:0009425 GO:0071973
tr|G8PPZ0|G8PPZ0_PSEUV	80	Flagellar biosynthetic protein FliP	fliP PSE_2611	GO:0005886 GO:0009306 GO:0009425 GO:0016021 GO:0044781
tr|A0A165N3N7|A0A165N3N7_9RHOB	79.2	Flagellar basal-body rod protein FlgG (Distal rod protein)	flgG_4 PsW74_05338	GO:0009426 GO:0071973
tr|A0A161XBY4|A0A161XBY4_9RHOB	79.1	Flagellar biosynthesis protein FlhA	flhA_2 PsAD26_02661	GO:0009306 GO:0016021 GO:0044780

tr|A0A165XJH0|A0A165XJH0_9RHOB	78.4	Flagellar biosynthetic protein FliQ	fliQ_2 PsAD2_02881	GO:0009306 GO:0016021 GO:0044780
tr|A0A0M6ZB61|A0A0M6ZB61_9RHOB	73.6	Flagellar FliL protein	fliL LA5096_00596	GO:0006935 GO:0009425 GO:0071973
tr|A0A165UF69|A0A165UF69_9RHOB	71.6	Flagellar transcriptional regulator FtcR	ftcR PsAD26_02623	GO:0000160 GO:0003677 GO:0005622 GO:0006351 GO:0006355
tr|A0A166C0M9|A0A166C0M9_9RHOB	69.9	Flagellar L-ring protein (Basal body L-ring protein)	flgH_2 flgH PsWM33_03307	GO:0003774 GO:0009279 GO:0009427 GO:0071973
tr|A0A0U3PKT9|A0A0U3PKT9_9RHOB	68.4	Flagellar biosynthesis repressor FlbT	flbT APZ00_14970	GO:0006402 GO:0048027 GO:1902209
tr|G8PP72|G8PP72_PSEUV	68.1	Flagellar basal-body rod protein FlgF	PSE_2594	GO:0030694 GO:0071973
tr|G8PP78|G8PP78_PSEUV	67.1	Flagellar biosynthesis protein FlhB	PSE_2600	GO:0009306 GO:0016021 GO:0044780
tr|A0A165UFX0|A0A165UFX0_9RHOB	62.6	Flagellar biosynthesis regulatory protein FlaF	PsAD26_02657	GO:0044781
tr|A0A165P2N7|A0A165P2N7_9RHOB	61.9	Flagellar basal body rod protein FlgB	PsW74_03890	GO:0071973
tr|A0A165N427|A0A165N427_9RHOB	61.4	Flagellar hook protein FlgE	flgE_2 PsW74_05352	GO:0005198 GO:0009424 GO:0009425 GO:0044780 GO:0071973
tr|A0A0U3PKT9|A0A0U3PKT9_9RHOB	68.4	Flagellar biosynthesis repressor FlbT	flbT APZ00_14970	GO:0006402 GO:0048027 GO:1902209
tr|A0A165UFG3|A0A165UFG3_9RHOB	59.4	Flagellar hook-basal body complex protein FliE	fliE_1 fliE PsAD26_02637	GO:0003774 GO:0005198 GO:0009425 GO:0071973
tr|A0A165MSM2|A0A165MSM2_9RHOB	59.3	Swarming motility protein SwrC	swrC_3 PsW74_05753	GO:0005215 GO:0016021
tr|B6R0C9|B6R0C9_9RHOB	55.1	Flagellar biosynthetic protein FliR	fliR PJE062_4556	GO:0005622 GO:0005886 GO:0006605 GO:0009425

				GO:0016021 GO:0044780
tr|G8PP84|G8PP84_PSEUV	50.3	Flagella basal body P-ring formation protein FlgA	PSE_2606	GO:0042597 GO:0044781
tr|B6R0B5|B6R0B5_9RHOB	45.4	Flageller protein FlgA, putative	PJE062_4669	-
tr|G8PQ01|G8PQ01_PSEUV	47.8	Flagellar hook-associated protein FlgK	flgK PSE_2622	GO:0005198 GO:0009424 GO:0044780
tr|A0A165N427|A0A165N427_9RHOB	61.4	Flagellar hook protein FlgE	flgE_2 PsW74_05352	GO:0005198 GO:0009424 GO:0009425 GO:0044780 GO:0071973
tr|A0A166ANU6|A0A166ANU6_9RHOB	42.4	Flagellar hook-associated protein FlgL	PsAD2_00653	-
tr|A0A166DHG0|A0A166DHG0_9RHOB	49	Flagellar hook-length control protein FliK	PsWM33_02218	GO:0005975 GO:0016810
tr|G8PP78|G8PP78_PSEUV	67.1	Flagellar biosynthesis protein FlhB	PSE_2600	GO:0009306 GO:0016021 GO:0044780
tr|A0A166ANC2|A0A166ANC2_9RHOB	33.8	Flagellar biosynthesis protein, FliO	PsAD2_00632	GO:0016021 GO:0044781

Supplementary File:
Biosurfactant: Classical pathway of fatty acid synthesis.

**Protein ID (Subject ID)**	**% Identity**	**Protein Names**	**Gene name**	**Gene Ontology IDs**
**Biosurfactant: Classical pathway of fatty acid synthesis**				
tr|B6R5C9|B6R5C9_9RHOB	77.6	Acetyl-coenzyme A carboxylase carboxyl transferase subunit alpha (ACCase subunit alpha) (Acetyl-CoA carboxylase carboxyltransferase subunit alpha) (EC 6.4.1.2)	accA PJE062_3339	GO:0003989 GO:0005524 GO:0006633 GO:0009317 GO:0016740 GO:2001295
tr|B6QXG9|B6QXG9_9RHOB	90.3	Acetyl-CoA carboxylase, biotin carboxylase (EC 6.4.1.2)	accC_1 PJE062_1664	GO:0003989 GO:0004075 GO:0005524 GO:0046872
tr|A0A165R1H4|A0A165R1H4_9RHOB	65.8	Biotin carboxyl carrier protein of acetyl-CoA carboxylase	accB PsW74_02842	GO:0003989 GO:0006633 GO:0009317
tr|A0A165R7R7|A0A165R7R7_9RHOB	90.2	Putative propionyl-CoA carboxylase beta chain 5 (EC 6.4.1.3)	accD5 PsW74_02264	GO:0004658
tr|G8PJE6|G8PJE6_PSEUV	85	Acetyl-coenzyme A carboxylase carboxyl transferase subunit beta (ACCase subunit beta) (Acetyl-CoA carboxylase carboxyltransferase subunit beta) (EC 6.4.1.2)	accD PSE_0340	GO:0003989 GO:0005524 GO:0006633 GO:0009317 GO:0016740 GO:2001295
tr|A0A166ECN3|A0A166ECN3_9RHOB	85.8	3-hydroxyacyl-[acyl-carrier-protein] dehydratase FabZ (EC 4.2.1.59) ((3R)-hydroxymyristoyl-[acyl-carrier-protein] dehydratase) (Beta-hydroxyacyl-ACP dehydratase)	fabZ PsWM33_01204	GO:0005737 GO:0006633 GO:0009245 GO:0047451
tr|B6R617|B6R617_9RHOB	83.5	3-oxoacyl-(Acyl-carrier-protein) synthase I (EC 2.3.1.41)	fabB PJE062_3309	GO:0004315 GO:0008152
tr|G8PJC8|G8PJC8_PSEUV	83.4	3-hydroxydecanoyl-[acyl-carrier-protein] dehydratase (EC 4.2.1.59) (3-hydroxyacyl-	fabA PSE_0322	GO:0005737 GO:0006633 GO:0008693

		[acyl-carrier-protein] dehydratase FabA) (Beta-hydroxydecanoyl thioester dehydrase) (Trans-2-decenoyl-[acyl-carrier-protein] isomerase)		GO:0034017 GO:0047451
tr|B6R7T4|B6R7T4_9RHOB	83	3-oxoacyl-[acyl-carrier-protein] synthase 2 (EC 2.3.1.179)	fabF_2 PJE062_4246	GO:0006633 GO:0033817
tr|B6QX84|B6QX84_9RHOB	82.8	3-oxoacyl-[acyl-carrier-protein] synthase 3 (EC 2.3.1.180) (3-oxoacyl-[acyl-carrier-protein] synthase III) (Beta-ketoacyl-ACP synthase III)	fabH PJE062_1033	GO:0004315 GO:0005737 GO:0006633 GO:0033818
tr|A0A0M6Y735|A0A0M6Y735_9RHOB	80.7	Enoyl-[acyl-carrier-protein] reductase [NADH] (EC 1.3.1.9)	fabI_2 LAL4801_03534	GO:0004318 GO:0006633
tr|A0A165UL10|A0A165UL10_9RHOB	79.6	3-oxoacyl-[acyl-carrier-protein] reductase FabG (EC 1.1.1.100)	fabG_5 PsAD2_03796	GO:0004316 GO:0006633 GO:0051287 GO:0102132
tr|A0A165ZMT9|A0A165ZMT9_9RHOB	75.6	Enoyl-[acyl-carrier-protein] reductase [NADH] (EC 1.3.1.9)	fabI_1 PsAD2_01557	GO:0004318 GO:0006633
tr|A0A165RW41|A0A165RW41_9RHOB	70	Malonyl CoA-acyl carrier protein transacylase (EC 2.3.1.39)	fabD PsW74_01680	GO:0004314 GO:0008152
tr|A0A0R3DSU7|A0A0R3DSU7_9BRAD	47.7	Enoyl-[acyl-carrier-protein] reductase [NADH] (ENR) (EC 1.3.1.9)	fabV AOQ73_02775	GO:0004318 GO:0006633 GO:0051287
tr|A0A0L8AB93|A0A0L8AB93_9SPHN	47.2	Enoyl-[acyl-carrier-protein] reductase [NADH] (ENR) (EC 1.3.1.9)	fabV W7K_08880	GO:0004318 GO:0006633 GO:0051287
**dTDP-L-rhamnose**				
tr|A0A165V1V1|A0A165V1V1_9RHOB	69.5	Rhamnolipids biosynthesis 3-oxoacyl-[acyl-carrier-protein] reductase (EC 1.1.1.100)	rhlG_1 PsAD26_01996	GO:0004316 GO:0102132
tr|A0A165QSK6|A0A165QSK6_9RHOB	68.7	dTDP-4-dehydrorhamnose reductase (EC 1.1.1.133)	rmlD PsW74_02537	GO:0008831
tr|Q0FS16|Q0FS16_PELBH	65.4	Rhamnulose-1-phosphate aldolase (EC 4.1.2.19)	R2601_17454	GO:0008994
tr|A0A165N228|A0A165N228_9RHOB	54.7	2-keto-3-deoxy-L-rhamnonate aldolase (EC 4.1.2.53)	rhmA PsW74_05287	GO:0016829 GO:0046872

tr|A5PAQ6|A5PAQ6_9SPHN	34	dTDP-4-dehydrorhamnose reductase	ED21_21564	-
**Acyl transferase**				
tr|U7GAR3|U7GAR3_9RHOB	64.4	Glycerol acyltransferase	Q669_05200	GO:0008152 GO:0016746
tr|A0A161VBE1|A0A161VBE1_9RHOB	62.3	1-acyl-sn-glycerol-3-phosphate acyltransferase (EC 2.3.1.51)	plsC PsAD26_00183	GO:0003841 GO:0008152 GO:0016021
tr|A0A166BAG3|A0A166BAG3_9RHOB	60.9	N-acyltransferase YncA (EC 2.3.1.-)	yncA_1 PsAD2_00092	GO:0008080
tr|A0A165UNQ8|A0A165UNQ8_9RHOB	62.4	Glycosyltransferase family 17	PsAD2_03927	GO:0003830 GO:0006487 GO:0016020
tr|A0A165XBP3|A0A165XBP3_9RHOB	54.8	Lipid A biosynthesis lauroyl acyltransferase	PsAD2_03087	GO:0016021 GO:0016746
tr|A0A166EXQ3|A0A166EXQ3_9RHOB	47.5	Apolipoprotein N-acyltransferase (ALP N-acyltransferase) (EC 2.3.1.-)	lnt PsWM33_00598	GO:0005886 GO:0006807 GO:0016021 GO:0016410 GO:0016810 GO:0042158
**Phosphomannomutase**				
tr|A0A165Y502|A0A165Y502_9RHOB	66.4	Alginate biosynthesis protein AlgA	algA PsWM33_04874	GO:0000271 GO:0016779
tr|B6R504|B6R504_9RHOB	81.6	Phosphomannomutase/phosphoglucomutase (EC 5.4.2.2) (EC 5.4.2.8)	algC PJE062_127	GO:0004614 GO:0004615 GO:0005975
tr|G8PSD0|G8PSD0_PSEUV	79.6	Phosphopentomutase (EC 5.4.2.7) (Phosphodeoxyribomutase)	deoB PSE_1044	GO:0000287 GO:0005737 GO:0006015 GO:0008973 GO:0009264 GO:0030145 GO:0043094
tr|A0A165PU10|A0A165PU10_9RHOB	77.6	Phosphoglucosamine mutase (EC 5.4.2.10)	glmM PsAD26_05283	GO:0000287 GO:0005975 GO:0008966
tr|A0A0P1J6R1|A0A0P1J6R1_9RHOB	71.1	UDP-galactopyranose mutase (EC 5.4.99.9)	rfbD_1 TA5114_01633	GO:0008767
tr|A0A086MG05|A0A086MG05_9RHIZ	67.3	N5-carboxyaminoimidazole ribonucleotide mutase (N5-CAIR mutase) (EC 5.4.99.18) (5-(carboxyamino)imidazole	purE JH26_20215	GO:0006189 GO:0034023

		ribonucleotide mutase)		
tr|B6R008|B6R008_9RHOB	67	Phosphoglycerate mutase family protein	PJE062_4716	-
tr|F2IYC6|F2IYC6_POLGS	63.5	Phosphoglycerate mutase family protein	gpmB SL003B_3316	-
tr|A0A165NSK3|A0A165NSK3_9RHOB	49.7	Methylmalonyl-CoA mutase small subunit (EC 5.4.99.2)	mutA PsW74_04152	GO:0004494 GO:0008152 GO:0031419 GO:0046872
**ACP (acyl-carrier-protein) reductase**				
tr|A0A165V1V1|A0A165V1V1_9RHOB	69.5	Rhamnolipids biosynthesis 3-oxoacyl-[acyl-carrier-protein] reductase (EC 1.1.1.100)	rhlG_1 PsAD26_01996	GO:0004316 GO:0102132
tr|A0A0F4RR42|A0A0F4RR42_9RHOB	68.3	3-ketoacyl-ACP reductase	TW80_00870	GO:0008678 GO:0051287
**ACP acyltransferase**				
tr|G8PLJ5|G8PLJ5_PSEUV	76.1	Phosphate acyltransferase (EC 2.3.1.n2) (Acyl-ACP phosphotransacylase) (Acyl-[acyl-carrier-protein]--phosphate acyltransferase) (Phosphate-acyl-ACP acyltransferase)	plsX PSE_3572	GO:0004147 GO:0005737 GO:0006633 GO:0008654 GO:0008951 GO:0016406 GO:0016411 GO:0016412 GO:0016413 GO:0016414 GO:0016416 GO:0016418 GO:0016419 GO:0016454 GO:0016616 GO:0016749 GO:0016750 GO:0016751 GO:0016753 GO:0018030 GO:0018031 GO:0018711 GO:0018712 GO:0018713

				GO:0019186<br>GO:0019705<br>GO:0032216<br>GO:0034737<br>GO:0034738<br>GO:0034848<br>GO:0034851<br>GO:0034915<br>GO:0034919<br>GO:0034945<br>GO:0043741<br>GO:0043806<br>GO:0043849<br>GO:0046941<br>GO:0052858<br>GO:0090595
tr|G8PKL2|G8PKL2_PSEUV	74.9	Beta-ketoadipyl-CoA thiolase (EC 2.3.1.16)	pcaF PSE_2283	GO:0003988<br>GO:0019619
tr|A0A166B4U6|A0A166B4U6_9RHOB	68.4	Acyl-[acyl-carrier-protein]--UDP-N-acetylglucosamine O-acyltransferase (UDP-N-acetylglucosamine acyltransferase) (EC 2.3.1.129)	lpxA PsAD2_00317	GO:0005737<br>GO:0008780<br>GO:0009245
tr|B6QXP3|B6QXP3_9RHOB	66.1	UDP-3-O-acylglucosamine N-acyltransferase (EC 2.3.1.-)	lpxD PJE062_1717	GO:0009245<br>GO:0016410
tr|A0A161X8U4|A0A161X8U4_9RHOB	65.4	Glycerol-3-phosphate acyltransferase (Acyl-PO4 G3P acyltransferase) (Acyl-phosphate--glycerol-3-phosphate acyltransferase) (G3P acyltransferase) (Lysophosphatidic acid synthase)	plsY_1 plsY PsAD2_04219	GO:0005886<br>GO:0008654<br>GO:0016021<br>GO:0043772
tr|A0A165RW41|A0A165RW41_9RHOB	70	Malonyl CoA-acyl carrier protein transacylase (EC 2.3.1.39)	fabD PsW74_01680	GO:0004314<br>GO:0008152
**ACP synthase**				
tr|B6R617|B6R617_9RHOB	83.5	3-oxoacyl-(Acyl-carrier-protein) synthase I (EC 2.3.1.41)	fabB PJE062_3309	GO:0004315<br>GO:0008152
